# Variation in blood pressure and long-term risk of dementia: A population-based cohort study

**DOI:** 10.1371/journal.pmed.1002933

**Published:** 2019-11-12

**Authors:** Yuan Ma, Frank J. Wolters, Lori B. Chibnik, Silvan Licher, M. Arfan Ikram, Albert Hofman, M. Kamran Ikram

**Affiliations:** 1 Department of Epidemiology, Erasmus MC University Medical Center, Rotterdam, the Netherlands; 2 Department of Epidemiology, Harvard T.H. Chan School of Public Health, Boston, Massachusetts, United States of America; 3 Department of Neurology, Erasmus MC University Medical Center, Rotterdam, the Netherlands; Guys & St Thomas’ NHS Foundation Trust, UNITED KINGDOM

## Abstract

**Background:**

Variation in blood pressure may relate to dementia risk via autonomic disturbance or hemodynamic mechanisms, but the long-term associations are unclear. We aimed to determine whether blood pressure variation over a period of years, considering both magnitude and direction, is associated with the risk of dementia.

**Methods and findings:**

In a prospective cohort study ongoing since 1989 in the Netherlands, 5,273 dementia-free participants (58.1% women; mean [SD] age, 67.6 [8.0] years) were included. As of 2016, 1,059 dementia cases occurred during a median follow-up of 14.6 years. Absolute variation in systolic blood pressure (SBP) was assessed as the absolute difference in SBP divided by the mean over two sequential visits every 4.2 (median) years, with the first quantile set as the reference level. The direction was the rise or fall in SBP, with the third quantile set as the reference level. We estimated the risk of dementia in relation to SBP variation measured at different time windows (i.e., at least 0, 5, 10, and 15 years) prior to dementia diagnosis, with adjustments for age, sex, education, apolipoprotein E (*APOE*) genotype, vascular risk factors, and history of cardiovascular disease. We repeated the above analysis for variation in diastolic blood pressure (DBP).

A large SBP variation was associated with an increased dementia risk, which became more pronounced with longer intervals between the assessment of SBP variation and the diagnosis of dementia. The hazard ratio (HR) associated with large variation (the highest quintile) increased from 1.08 (95% confidence interval [CI] 0.88–1.34, *P* = 0.337) for risk within 5 years of SBP variation measurement to 3.13 (95% CI 2.05–4.77; *P* < 0.001) for risk after at least 15 years since the measurement of SBP variation. The increased long-term risk was associated with both large rises (HR for the highest quintile, 3.31 [95% CI 2.11–5.18], *P* < 0.001) and large falls in SBP (HR for the lowest quintile, 2.20 [95% CI 1.33–3.63], *P* = 0.002), whereas the higher short-term risk was only associated with large falls in SBP (HR, 1.21 [95% CI 1.00–1.48], *P* = 0.017). Similar findings were observed for variation in DBP. Despite our assessment of major confounders, potential residual confounding is possible, and the findings on blood pressure variability over periods of years may not be generalizable to variability over periods of days and other shorter periods.

**Conclusions:**

Results of this study showed that a large blood pressure variation over a period of years was associated with an increased long-term risk of dementia. The association between blood pressure variation and dementia appears most pronounced when this variation occurred long before the diagnosis. An elevated long-term risk of dementia was observed with both a large rise and fall in blood pressure.

## Abstract 2

Albert Hofman and colleagues investigate associations between individual blood pressure changes and dementia risk.

## Author summary

**Why was this study done?:**

- Blood pressure rises throughout most of life, and hypertension is proposed as an important modifiable risk factor for dementia. In late life, the relationship between blood pressure and dementia is controversial.
- Variation in blood pressure is suggested to be associated with dementia risk beyond absolute blood pressure levels, but the long-term associations are unclear. Whether the putative associations differ by direction of variation is also unknown.

**What did the researchers do and find?:**

- In this prospective cohort study involving 5,273 dementia-free participants who were followed over 26 years, a large blood pressure variation, measured over two sequential visits every 4 years apart, was associated with an increased risk of dementia, especially when this variation occurred long before the diagnosis.
- Both large rises and falls in blood pressure were associated with a higher long-term risk.

**What do these findings mean?:**

- This study suggests the potential importance of blood pressure variability in the etiology of dementia.
- If the observed association is causal, our study suggests an opportunity to prevent dementia through targeting large variation in blood pressure over a period of years above and beyond the mere control of conventional blood pressure limits.
- The stronger association over longer intervals thereby suggests greater benefits from interventions implemented earlier in life.

## Introduction

Dementia is the most common neurodegenerative disease in elderly people, associated with high disability and dependency [1]. Around 50 million people are living with dementia globally, and because of the aging population, the number of patients is predicted to triple by 2050, with global economic costs projected to rise in parallel [1,2].

Vascular risk factors are potentially major modifiable contributors in the multifactorial etiology of cognitive decline and dementia [3]. Hypertension is a particularly important risk factor, but its relation to dementia becomes complex with aging [4,5]. This association may depend on time until diagnosis [6], and dynamic effects of blood pressure fluctuation are unlikely to be captured in a single measurement. Blood pressure variability emerges as a risk factor for ischemic stroke, with effects beyond absolute blood pressure levels alone [7,8]. It has also been reported that a larger variability in systolic blood pressure (SBP) and diastolic blood pressure (DBP) over time is associated with a higher risk of dementia during a follow-up of up to 8 years [9]. Given the insidious onset of dementia, pathological processes of dementia affecting blood pressure may occur many years before the diagnosis, and short-term associations may thus be susceptible to reverse causation [10]. It is unknown whether blood pressure variation is associated with dementia in the long-term and whether the putative association changes over time. Moreover, there is no consistent evidence on whether the direction of variation—i.e., rise or fall in blood pressure—is relevant to subsequent dementia risk. The mechanisms underlying the putative associations, possibly involving vascular stiffness [11], also remain undetermined.

We investigated the association between blood pressure variation and the risk of dementia in a prospective cohort study with up to 26 years of follow-up, considering both magnitude and direction of the variation measured at a range of time intervals prior to the diagnosis of dementia.

## Methods

### Ethics statement

The Rotterdam Study has been approved by the institutional review board (Medical Ethics Committee) of the Erasmus Medical Center and by the review board of The Netherlands Ministry of Health, Welfare and Sport. Written informed consent has been obtained from all participants.

### Study design and data sources

This study is embedded in the Rotterdam Study, a prospective cohort study underway since 1989 in the Ommoord District in the city of Rotterdam, the Netherlands. A detailed description has been published elsewhere [12]. Data were collected following a prospective study protocol [12]. Statistical analyses were performed following a prospective analysis plan with prespecified research hypothesis (S1 Text). Briefly, 7,983 participants (out of 10,215 invitees) aged ≥55 years have been followed for 26 years (since July 27, 1989, through January 1, 2016), with the first through fifth examination cycles performed in 1989–1993, 1993–1995, 1997–1999, 2002–2004, and 2009–2011. The present study includes all participants free of dementia at the first and second examination cycles. We applied the following exclusion criteria: insufficient data on dementia status at the first visit (*n* = 348), prevalent dementia at the first visit (*n* = 486), no informed consent for follow-up data collection (*n* = 100), incident dementia before the second visit (*n* = 404), death before completing at least two visits (*n* = 1,034), and missing blood pressure measurements at the first two visits (*n* = 338). Ultimately, 5,273 participants were eligible for the current study (Fig A in S2 Text). A comparison of eligible versus ineligible participants is provided in the Supporting information (Table A in S2 Text). Eligible participants were generally younger, had a lower risk profile for vascular disease, and had better cognitive function at baseline.

### Variation in blood pressure

At each visit, after at least 5 minutes’ rest in a seated position, two blood pressure measurements were taken on the right upper arm. The mean of these two measurements was used for that visit. Blood pressure was measured in the same way from the first through the fifth visits. Before November 7, 2006, a Hawksley random-zero sphygmomanometer was used. Omron M6 Comfort and Omron M7 devices were used thereafter. We assessed variation in SBP, DBP, and pulse pressure separately and primarily reported results on variability in SBP because of the stronger association of SBP with adverse health outcomes [13].

**Fig 1:**
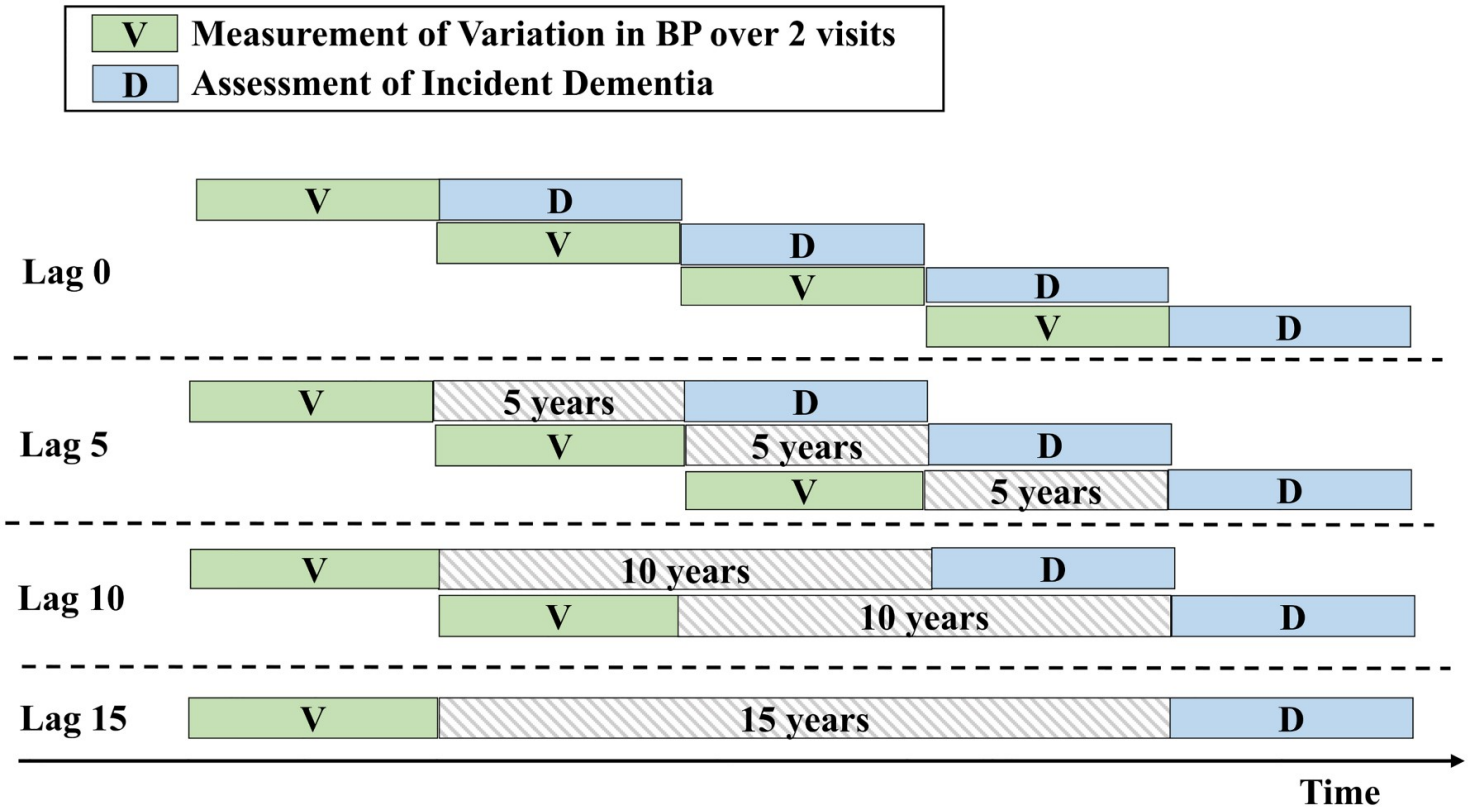
Schematic diagram of the analyses relating BP variation to the risk of dementia at different lag periods. ^a^The years of lag indicate the minimum interval between the measurement of BP variation and the assessment of incident dementia. A lag of 0 examined the risk of dementia during the visit interval immediately following the measurement of BP variation. A lag of 5 years investigated the risk of dementia occurring at least 5 years after the measurement of BP variation, in which dementia cases occurring within the first 5 years of follow-up were censored, and so on. BP, blood pressure.

Within-individual SBP variation between two sequential visits was assessed at the latter of the two visits using two measures: (1) variation in SBP as the primary measure to capture the magnitude of variation and (2) directional variation as a secondary measure to differentiate rises from falls in SBP. The variation was calculated as the absolute difference in SBP divided by the mean SBP over two sequential visits (|difference|/mean). Similarly, directional variation was defined as the difference in SBP between the two visits divided by the mean ([latter − former]/mean). We assessed SBP variation over a rolling time window of two sequential visits because it allowed us to better examine lag-specific associations and to differentiate the direction of SBP variation. To account for different visit intervals (median, 4.2; 25th–75th percentile, 2.0–4.8 years), both measurements were scaled to the average variation per year, assuming a constant rate of variation between the two visits. As shown in Fig 1, measurements were assessed as time-varying exposures, first assessed at the second visit using SBP of the first two visits, and then updated at the third visit using SBP of the second and third visits, and so on. Of 5,273 participants, 5,088 had valid SBP measured at consecutive visits before censoring, with the number of visits with SBP measurements ranging from 2 to 5 per participant. For the 185 (3.5%) participants who missed one visit in the middle, variation assessed at the previous visit was used. The median number of visits with SBP measurements used for each lag analysis was 4 (lag 0), 4 (lag 5), 4 (lag 10), and 3 (lag 15) per participant.

### Ascertainment of dementia

Participants were screened for dementia at baseline and subsequent visits using the Mini-Mental State Examination and the Geriatric Mental Schedule. Participants having a Mini-Mental State Examination score < 26 or Geriatric Mental State Schedule organic level > 0 underwent further examination, including the Cambridge Examination for Mental Disorders of the Elderly. All participants also underwent routine cognitive assessment. Additionally, all participants were continuously monitored for dementia through electronic linkage with medical records from general practitioners and the regional institute for outpatient mental healthcare. Available information on cognitive testing and clinical neuroimaging was used when required for diagnosis of dementia subtype. A consensus panel led by a consultant neurologist established the final diagnosis according to standard criteria for dementia (the *Diagnostic and Statistical Manual of Mental Disorders*, *Third Edition*, *Revised*; *DSM-III-R*), Alzheimer’s disease (NINCDS-ADRDA), and vascular dementia (NINDS-AIREN) [14].

### Covariates

Information on demographic characteristics was collected at the first visit. The apolipoprotein E (*APOE*) genotype was determined using polymerase chain reaction on coded genomic DNA samples. During each visit, smoking habits, alcohol consumption, medication use, body mass index, total cholesterol, high-density lipoprotein cholesterol, and diabetes mellitus were assessed with standardized protocols. Antihypertensive medication was classified according to WHO Anatomical Therapeutic Chemical Codes and included antihypertensives (C02), diuretics (C03), beta blockers (C07), calcium channel blockers (C08), and renin-angiotensin system modifying agents (C09). Cardiovascular disease, including stroke, coronary heart disease, heart failure, and atrial fibrillation, was assessed via interviews and verified by medical records. Arterial stiffness was assessed by carotid–femoral pulse wave velocity at the third visit (*n* = 3,191) using an automatic device (S3 Text).

### Statistical methods

#### Primary analyses

Our analysis focused on the association between variation in SBP, assessed over two sequential study visits, and incident dementia. Person-time accrued from the second visit (first assessment of SBP variation) until the date of dementia diagnosis, date of death, date of loss to follow-up, or administrative censoring on January 1, 2016, whichever came first (with near-complete follow-up of 98% of potential person-years). Given the potentially long but unclear latency period for dementia, we performed analyses with varied latency periods. As shown in Fig 1, we estimated the associations considering four lag periods, defined as a lag period of 0, 5, 10, and 15 years, respectively. The years of lag represent the minimum interval between the measurement of SBP variation and the assessment of incident dementia. Specifically, a lag of 0 years investigated new dementia cases occurring during the visit interval immediately following the measurement of SBP variation. A lag of 5 years investigated new dementia cases occurring at least 5 years after the measurement of SBP variation, and so on. In the lag 5 analysis, individuals with a follow-up of less than 5 years were not included, because they did not have measurements on SBP variation at the specified time windows. Likewise, individuals with a follow-up of less than 10 years were not included in the lag 10 analysis, and so on. Cox models with time-dependent covariates were used to estimate hazard ratios (HR) for incident dementia. The corresponding HRs with longer lags reflect incrementally longer-term associations. Inverse-probability weights were employed in all the primary analyses to reduce potential selection bias, with additional information provided (S3 Text) [15].

For the analysis of each of the lag windows (i.e., for lag 0, lag 5, lag 10, and lag 15), the continuous measure of SBP variation was divided into five categories by the quintiles of all measurements of SBP variation, with the reference group defined as the lowest quintile for absolute variation and the middle quintile for directional variation. Testing for linear trends across quintiles of variation in SBP was performed by entering a single ordinal term. We additionally examined the associations with SBP variation using restricted cubic-spline term to assess the deviation from linearity [16]. The change in the association over lag periods was further examined with 1-year increments in lag from 0 to 15 years.

To control for possible confounding that may affect both SBP variation and dementia risk, Cox models were built for each of the four time windows described above in the following three ways: (1) adjustment for age and sex; (2) additional adjustment for mean SBP; (3) adjustment for age, sex, education level, and *APOE* genotype, as well as time-dependent covariates, which were updated simultaneously with variation in SBP, including smoking habits, alcohol consumption, body mass index, lipid levels, history of diabetes and cardiovascular disease, and antihypertensive medication use at each of the two visits when SBP variation was assessed. All covariates, except SBP level and age, were categorical, and missing data were handled by adding an additional category indicating missing values (<10%). We also used a multiple-imputation approach with five imputations in our sensitivity analysis, which showed consistent results. Findings from the three models were similar, and therefore, results from the final model are presented.

To identify potential effect modification, we stratified the analyses by antihypertensive treatment during the study, SBP level at baseline, age, and sex. Interaction was formally tested on a multiplicative scale by adding a product term to the model. To explore potential mechanisms, we further stratified the association by pulse wave velocity index, the most common indicator of arterial stiffness [17].

#### Secondary analyses

We repeated the above analyses for the most common subtypes of dementia—i.e., Alzheimer’s disease and vascular dementia—for the variation in DBP and pulse pressure, and for SBP variation using absolute difference in SBP (mmHg per year), respectively. To account for the competing risk of death, we estimated cause-specific HRs for dementia and death, respectively [18]. To allow for the comparison with previous studies on this topic [9,19], we further examined (time-independent) SBP variation over the first three visits, spanning 6 years, assessed by coefficient of variation and standard deviation. The proportional hazard assumptions were tested by including an interaction term with time in the model, and the assumptions were also verified. The correlation between these measures was also assessed.

#### Sensitivity analyses

To test the robustness of the main findings, we performed the following analyses: (1, 2) excluding participants with a history of cardiovascular disease and diabetes mellitus, respectively; (3) censoring SBP measurements after the onset of cardiovascular disease; (4) restricting analyses to those with SBP measurements at consecutive visits before censoring; (5) reporting associations without using inverse-probability weighting; (6) imputing missing data using a multiple-imputation approach; (7) censoring participants at the diagnosis of stroke to assess the relationship that is not mediated by nonfatal stroke; and (8) estimating how strong residual confounding would need to be to explain away the observed associations [20].

This study is reported according to the Strengthening the Reporting of Observational Studies in Epidemiology (STROBE) guidelines (S1 STROBE Checklist). All effect estimates are given with corresponding 95% confidence intervals (CIs). All *P* values presented are two sided, with a *P* value of 0.05 or less considered statistically significant. Statistical analyses were performed using SAS version 9.4 (SAS Institute) and R version 3.4.2 (R Foundation).

## Results

### Study population

Of 5,273 participants, 3,063 (58.1%) were women, and the mean (SD) age was 67.6 (8.0) years. During a median follow-up of 14.6 years (from 1989 to 2016, interquartile range 7.9–20.5), 1,059 participants developed dementia (overall incidence rate: 14.7 cases per 1,000 person-years), including 802 (75.7%) with Alzheimer’s disease and 80 (7.6%) with vascular dementia. Table 1 describes the participant characteristics.

**Table 1:** Participant characteristics.

Characteristics ^a^	Overall (*n* = 5,273)
Age, years	67.6 ± 8.0
Women, *n* (%)	3,063 (58.1)
Education, *n* (%)	
Primary education only	1,037 (19.9)
Intermediate education	3,698 (70.8)
Higher vocation/university education	486 (9.3)
*APOE* genotype, *n* (%)	
ε3/ε3	2,957 (58.4)
ε2/ε2 or ε2/ε3	696 (13.8)
ε2/ε4 or ε3/ε4	1,294 (25.6)
ε4/ε4	113 (2.2)
Systolic blood pressure, mmHg	138 ± 22
Diastolic blood pressure, mmHg	74 ± 11
Pulse pressure, mmHg	64 ± 17
Hypertension, *n* (%)	3,100 (58.8)
Antihypertensive treatment at baseline, *n* (%)	1,544 (29.3)
Antihypertensive treatment during follow-up, *n* (%)	
Intermittent treatment	2,010 (38.1)
Continuous treatment	1,107 (21.0)
Weight status^b^, *n* (%)	
Overweight	2,465 (47.2)
Obese	762 (14.6)
Smoking status, *n* (%)	
Past	2,184 (42.9)
Current	1,057 (20.8)
Current alcohol drinking, *n* (%)	3,621 (80.8)
Body mass index, kg/m^2^	26.3 ± 3.6
Total cholesterol, mmol/L	6.7 ± 1.2
HDL cholesterol, mmol/L	1.4 ± 0.4
History of diabetes, *n* (%)	336 (6.7)
History of cardiovascular disease, *n* (%)	669 (12.7)
Stroke, *n* (%)	101 (1.9)
Coronary heart disease, *n* (%)	373 (7.1)
Atrial fibrillation, *n* (%)	203 (3.8)
Heart failure, *n* (%)	121 (2.3)

Data are shown in the format of mean ± SD and *n* (%).

^a^Characteristics at the first visit after cohort entry unless otherwise specified.

^b^Weight status was assessed by BMI, with overweight defined as 25 ≤ BMI < 30 kg/m^2^ and obesity defined as BMI ≥ 30 kg/m^2^.

Abbreviations: *APOE*, apolipoprotein E; BMI, body mass index; HDL, high-density lipoprotein

### Variation in SBP and the risk of dementia

**Fig 2:**
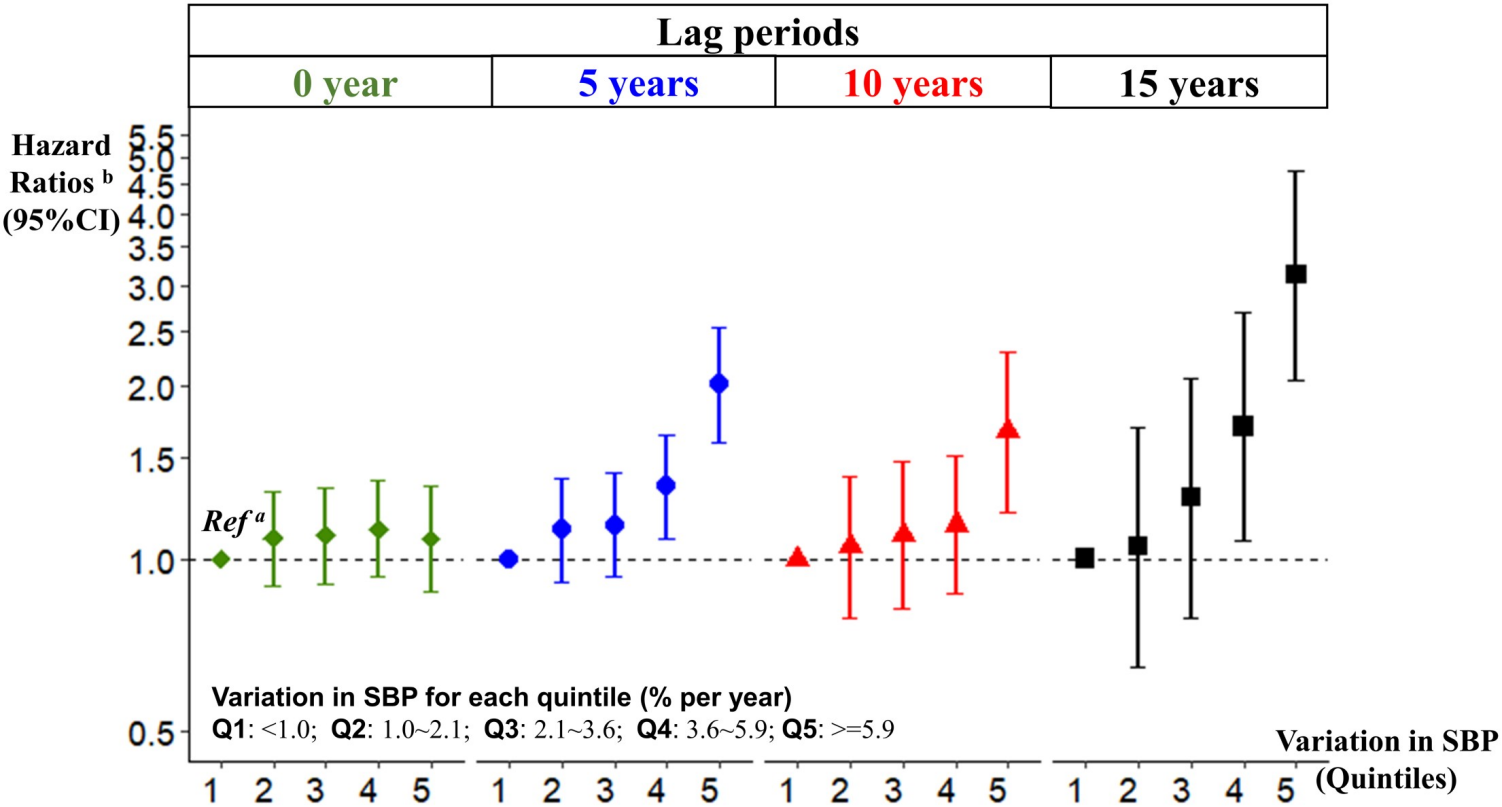
Variation in SBP and the risk of dementia. ^a^*Ref*. defined as the lowest quintile, representing the smallest variation in SBP. ^b^Adjusting for age, sex, education level, *APOE* genotype, and time-dependent covariates on smoking habits, alcohol consumption, use of antihypertensive medication, body mass index, lipid level, and history of diabetes and cardiovascular disease. *APOE*, apolipoprotein E; SBP, systolic blood pressure; CI, confidence interval; Q, quintile; *Ref*., reference level.

A large SBP variation was associated with a higher risk of dementia. Table 2 and Fig 2 show the HRs of dementia by quintiles of SBP variation after adjusting for age, sex, education, *APOE* genotype, vascular risk factors, and history of cardiovascular disease. In the short-term (lag0), the risk of dementia was not statistically significantly associated with SBP variation. The magnitude of the association increased with longer intervals between the measurement of SBP variation and dementia diagnosis, and the HR for a large variation was 3.13 (comparing highest versus lowest quintile; 95% CI 2.05–4.77, *P* < 0.001) when measured ≥15 years ago. The associations estimated for every 1-year increase in the lag period from 0 to 15 years demonstrate an upward trend, which reached statistical significance from a lag period of 1 year onwards (Fig B in S2 Text).

**Table 2:** Variation in systolic blood pressure and risk of dementia.

Lag periods (years)	Events/participants at risk	Hazard ratios (95% CI)^a^					
		Quintile 1^b^ (<1.0%/year)	Quintile 2 (1.0~2.1%/year)	Quintile 3 (2.1~3.6%/year)	Quintile 4 (3.6~5.9%/year)	Quintile 5 (≥5.9%/year)	*P* for linear trend
0	1,059/5,273	1	1.09 (0.90–1.31)	1.10 (0.91–1.33)	1.13 (0.93–1.37)	1.08 (0.88–1.34)	0.337
5	878/4,532	1	1.12 (0.91–1.38)	1.15 (0.93–1.42)	1.34 (1.08–1.65)	2.01 (1.60–2.54)	<0.001
10	586/3,472	1	1.05 (0.79–1.40)	1.10 (0.82–1.47)	1.15 (0.87–1.51)	1.67 (1.21–2.30)	0.006
15	360/2,565	1	1.05 (0.65–1.70)	1.28 (0.79–2.07)	1.70 (1.08–2.69)	3.13 (2.05–4.77)	<0.001

^a^With adjustment for age, sex, education level, *APOE* genotype, and time-dependent covariates on smoking habit, alcohol consumption, the use of antihypertensive medication, body mass index, lipid level, and history of diabetes and cardiovascular disease.

^b^Reference category.

Abbreviations: *APOE*, apolipoprotein E; CI, confidence interval

**Fig 3:**
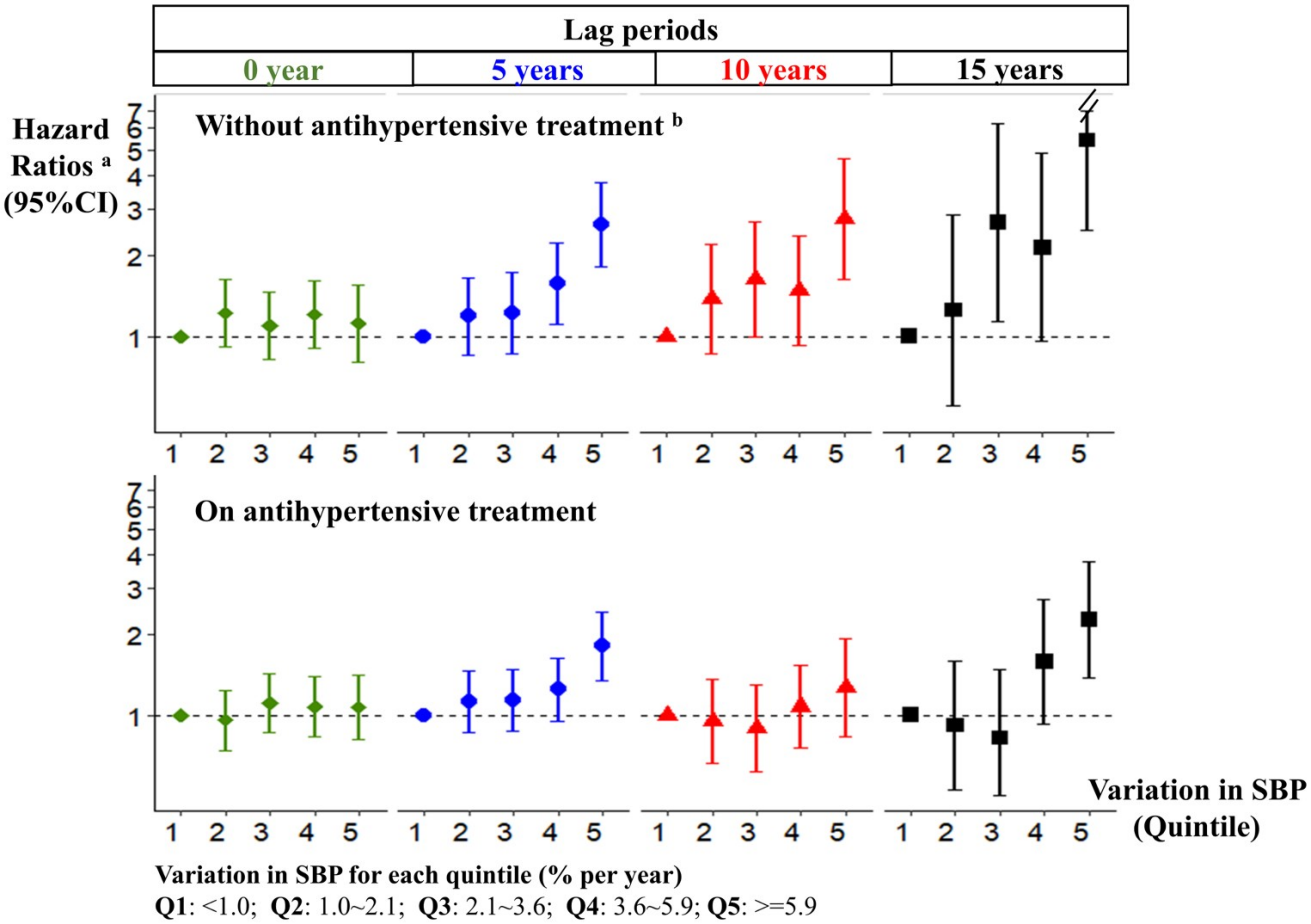
SBP variation and the risk of dementia by antihypertensive medication. ^a^Adjusting for age, sex, education level, *APOE* genotype, and time-dependent covariates on smoking habits, alcohol consumption, body mass index, lipid level, and history of diabetes and cardiovascular disease. ^b^*P* values for interaction term (between SBP variation and antihypertensive medication) were 0.68, 0.74, 0.13, and 0.02, with a lag period of 0, 5, 10, and 15 years, respectively. *APOE*, apolipoprotein E; SBP, systolic blood pressure; CI, confidence interval; Q, quintile.

A stronger association between SBP variation and dementia was noted especially over longer intervals in those not on antihypertensive treatment during the study (Fig 3). There is no clear difference in the association estimates according to baseline SBP level (Table B in S2 Text). The association appeared stronger in younger participants aged <70 years and in men (Table C in S2 Text). It also appeared stronger with the presence of arterial stiffness (Table D in S2 Text).

### Rise or fall in SBP and the risk of dementia

**Fig 4:**
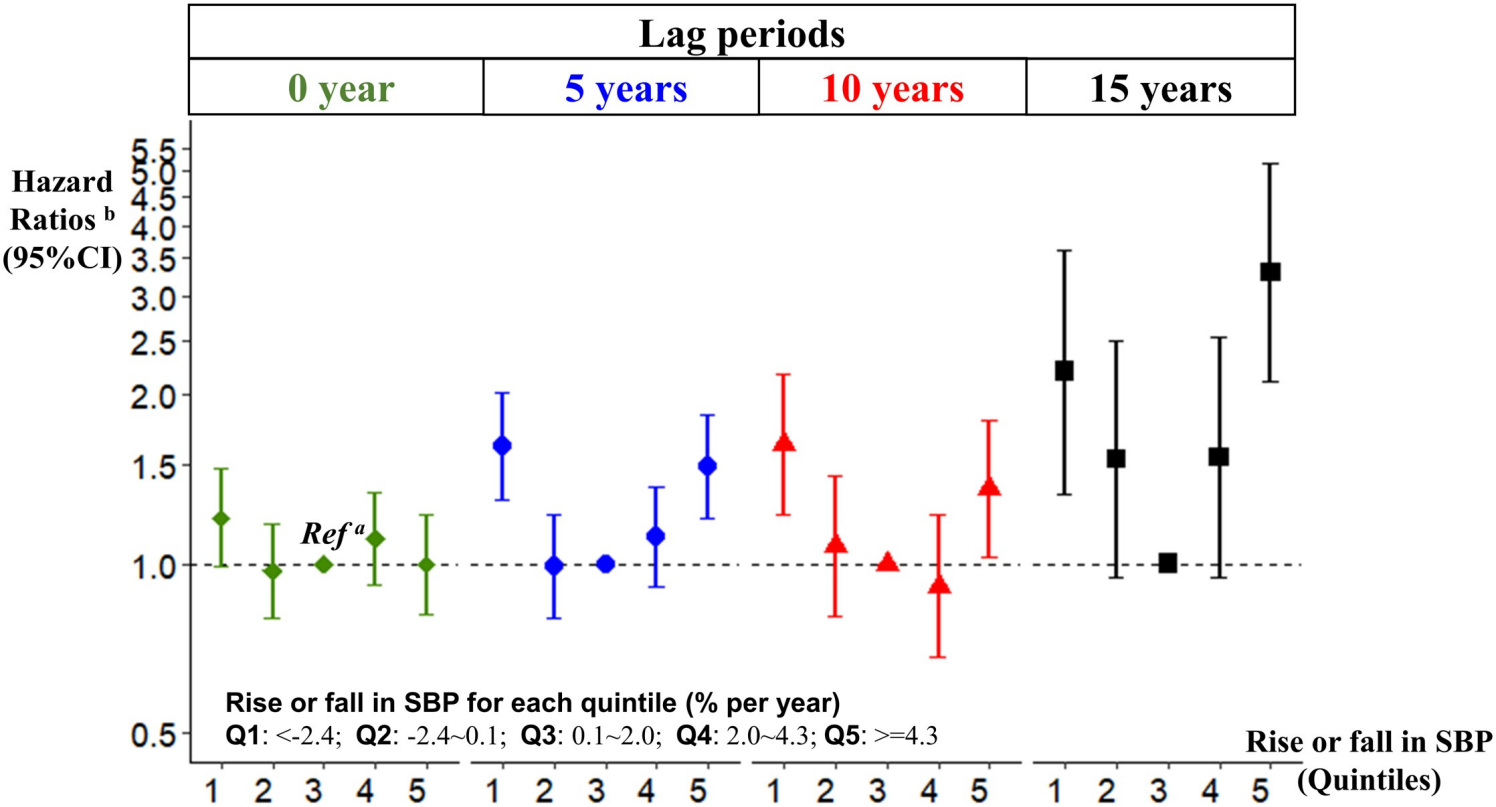
Rise or fall in SBP and the risk of dementia. ^a^*Ref*. defined as the third quintile, representing the smallest variation in SBP. ^b^Adjusting for age, sex, education level, *APOE* genotype, and time-dependent covariates on smoking habits, alcohol consumption, use of antihypertensive medication, body mass index, lipid level, and history of diabetes and cardiovascular disease. *APOE*, apolipoprotein E; SBP, systolic blood pressure; CI, confidence interval; Q, quintile; *Ref*., reference level.

Long-term associations of a large SBP variation with an increased dementia risk were observed for both large rises and large falls in SBP after adjusting for age, sex, education, *APOE* genotype, vascular risk factors, and history of cardiovascular disease (Fig 4, Table 3). This U-shaped association was enhanced with longer intervals. The HR was 3.31 (comparing highest versus middle quintile of variation in SBP; 95% CI 2.11–5.18, *P* < 0.001) with large rises in SBP and 2.20 (comparing lowest versus middle quintile; 95% CI 1.33–3.63, *P* = 0.002) with large falls in SBP occurring ≥15 years before diagnosis. Short-term associations differed from the long-term associations, showing modest increased risk of dementia only with large falls in SBP ≤5 years before diagnosis (lag0; HR, 1.21; 95% CI 1.00–1.48; *P* = 0.017. Fig 4, Table 3).

**Table 3:** Rise or fall in systolic blood pressure and risk of dementia.

Lag periods (years)	Events/participants at risk	Hazard ratios (95% CI)^a^					
		Quintile 1 (<−2.4%/year)	Quintile 2 (−2.4~0.1%/year)	Quintile 3^b^ (0.1~2.0%/year)	Quintile 4 (2.0~4.3%/year)	Quintile 5 (≥4.3%/year)	*P* for nonlinear trend^c^
0	1,059/5273	1.21 (1.00–1.48)	0.97 (0.80–1.18)	1	1.11 (0.92–1.34)	1.00 (0.81–1.22)	0.017
5	878/4,532	1.62 (1.30–2.01)	0.99 (0.80–1.22)	1	1.11 (0.91–1.37)	1.49 (1.20–1.84)	<0.001
10	586/3,472	1.63 (1.22–2.17)	1.08 (0.80–1.44)	1	0.91 (0.68–1.22)	1.36 (1.03–1.81)	<0.001
15	360/2,565	2.20 (1.33–3.63)	1.54 (0.94–2.50)	1	1.55 (0.94–2.53)	3.31 (2.11–5.18)	<0.001

^a^With adjustment for age, sex, education level, *APOE* genotype, and time-dependent covariates on smoking habit, alcohol consumption, the use of antihypertensive medication, body mass index, lipid level, and history of diabetes and cardiovascular disease.

^b^Reference category.

^c^Test for nonlinearity for the spline term of the rise or fall in systolic blood pressure.

Abbreviations: *APOE*, apolipoprotein E; CI, confidence interval

### Secondary analyses

The magnitudes of association with large SBP variation were somewhat larger for vascular dementia than for Alzheimer’s disease (Table E in S2 Text). Similar association was observed for variation in both DBP (Table F in S2 Text) and pulse pressure (Table G in S2 Text), though the association estimates for pulse pressure variation appeared smaller with less consistent patterns. The association with SBP variation measured by absolute difference—i.e. in mmHg per year—remained essentially unchanged (Table H in S2 Text). Cause-specific HRs estimated for dementia were consistent with primary findings, with similar association patterns observed for all-cause mortality (Table I in S2 Text). The association of the magnitude of variation in SBP from the first three visits over 6 years, measured by coefficient of variation and standard deviation, were essentially consistent with the primary findings (Table J in S2 Text). The correlation between these measures on SBP variation is also provided (Table K in S2 Text).

### Sensitivity analyses

Findings were consistent in all the sensitivity analyses (Table L in S2 Text). Specifically, association estimates appeared stronger after excluding individuals with cardiovascular disease and diabetes mellitus at baseline. A final sensitivity analysis showed that, to explain away dementia risk ≥5 years after the measurement of SBP variation (HR with a large variation in SBP, 2.01; 95% CI 1.60–2.54), the unmeasured confounding would need to be associated with both a large SBP variation and dementia by an HR of 3.43 each, above and beyond the measured confounders [20].

## Discussion

We found that a large blood pressure variation was associated with an increased risk of dementia in a 26-year prospective cohort study. The association appeared stronger as the years between the measurement of blood pressure variation and the diagnosis of dementia increased. A higher risk of dementia was observed with both large rises and falls in blood pressure, suggesting that a large variation in blood pressure, rather than the direction of the variation, increases the risk of dementia.

We observed an increased long-term risk of dementia associated with a large blood pressure variation over a period of years, independent of concurrent blood pressure level. This observation was in line with a previous study with a shorter follow-up [9]. Another study observed a significant association of large blood pressure variability with cognitive decline but not with incident dementia [19]. Together with the body of evidence linking blood pressure variation to cardiovascular disease and cognitive decline [8,21,22], our finding suggests that a large blood pressure variation over a period of years may be an important marker of impaired blood pressure regulation, especially in the aging population. Blood pressure rises throughout most of life, but in late life, blood pressure varies substantially, and a decline in both SBP and DBP has been observed [23,24]. The timing and determinants for the downward trend in blood pressure are also unclear [25]. By focusing on variation in blood pressure, this finding reconciles previous data linking not only large rises but also large falls in blood pressure to dementia [26–28].

The stronger association of blood pressure variation with dementia over longer intervals is consistent with the evidence showing that midlife hypertension is especially strongly associated with dementia [29]. This trend suggests a robust relationship that suffers less from reverse causation. It may also reflect a cumulative effect of chronic augmented fluctuation in cerebral blood flow. The magnitude of the association tended to be greater for vascular dementia than for Alzheimer’s disease, consistent with existing evidence [9,30]. The consistent and more profound associations, observed in the absence of antihypertensive treatment and among those of lower blood pressure, concur with previous reports [9,21,31]. This observation suggests that the observed association was not explained by the initiation of or the change in antihypertensive medication during the study.

The biological mechanisms underlying the association are largely unknown. A large blood pressure variation over a period of years, including both large rises and falls, could reflect the progression of vascular pathology or a progressive impairment of blood pressure regulation through multiple pathways. The results suggest a possibly more detrimental role of large blood pressure variation in the presence of elevated pulse wave velocity. One explanation could be that stiffness of large vessels may increase pulsation of flow and dampen the smoothing of blood flow as it progresses to small arteries, particularly in high-flow organs such as the brain [32,33]. Therefore, in the presence of arterial stiffness with advancing age, the exposure to wider pressure fluctuations, including large rises and falls in blood pressure, may damage the microvasculature of the brain and cause brain atrophy and cerebral small-vessel disease, thereby leading to dementia [34]. Age-related endothelial dysfunction could be another explanation. Animal studies suggest that large blood pressure variability could impair endothelial function by inhibiting nitric oxide production, contributing to “neurovascular unit” injuries and cerebral small-vessel disease [35,36].

The association of blood pressure variation with the short-term risk of dementia was moderate, as a higher risk was observed only with substantial falls in blood pressure. This observation was consistent with evidence linking late-life declines in blood pressure to dementia [26]. One explanation is that the lower cerebral autoregulation threshold is more likely to be impaired and shifts upwards during aging and with hypertension [37], thereby subjecting individuals with steep declines in blood pressure more vulnerable to cerebral hypoperfusion, a putative risk factor for dementia [38]. Alternatively, reverse causation is possible. Pathological changes of prodromal dementia may affect central autonomic regulation to stabilize blood pressure, resulting in a large variation, especially large falls due to the impairment of sympathetically mediated vasoconstriction [39].

Several limitations need to be acknowledged. First, we measured blood pressure variation over a period of 2–4 years, which is clearly different from short-term blood pressure variability over hours, days, and weeks. Assessing blood pressure variation using data from no more than two visits could attenuate risk estimates because of random measurement error. The consistent findings from different time windows and the strong dose-response associations indicate that the pathological changes underlying the variation spanning years were strong enough to manifest themselves even in the presence of random noise. Second, the physiological mechanisms underlying blood pressure variation over different time intervals are largely unknown, and our findings may therefore not be generalizable to diurnal, beat-to-beat, and day-to-day variation. Third, despite the use of inverse-probability weights, including only the surviving individuals in the analyses with longer lag periods may have introduced selection bias. Fourth, residual confounding is possible, although this is unlikely to change our conclusions, given the strength this unmeasured confounding would need to have to explain away the observed effect estimates. Finally, reverse causation, a much less likely issue for the long-term associations, is still possible if prodromal dementia starts 20 years before the diagnosis. This study has several strengths, including a continuous monitoring and standardized diagnosis of dementia and an investigation of the association across intervals ranging from 5 years to more than 15 years apart.

### Conclusions

In this study, we observed that a large variation in blood pressure was associated with an increased risk of dementia. The strength of the association between blood pressure variation and dementia appeared stronger with longer intervals. Given the paucity of epidemiological evidence on dementia from long-term prospective studies, this study may offer important insights into the etiology of dementia. If the observed association is causal, our study suggests a large potential to prevent dementia through targeting blood pressure variability above and beyond the mere control of conventional blood pressure limits—for instance, by the preferred use of calcium channel blockers and non-loop diuretics [40]. Future clinical trials for blood pressure control to prevent cognitive decline could therefore benefit from incorporating targets to maintain stable blood pressure over time. The stronger association over longer intervals thereby suggests greater benefits from interventions implemented earlier in life.

## Supporting information

S1 STROBE Checklist STROBE, Strengthening the Reporting of Observational Studies in Epidemiology. (DOC)

S1 Text Analysis plan. (PDF)

S2 Text Supplementary tables and figures. (DOCX)

S3 Text Detailed methodology. (DOCX)

## Abbreviations

*APOE*: apolipoprotein E
CI: confidence interval
DBP: diastolic blood pressure
*DSM-III-R*: *Diagnostic and Statistical Manual of Mental Disorders*, *Third Edition*, *Revised*
HR: hazard ratio
SBP: systolic blood pressure
STROBE: Strengthening the Reporting of Observational Studies in Epidemiology
