# Supplementary material for: Variation in blood pressure and long-term risk of dementia: A population-based cohort study
Source: PLoS Med. 2019 Nov 12;16(11):e1002933. doi: 10.1371/journal.pmed.1002933 (PMC6850672; doi:10.1371/journal.pmed.1002933)
Supplement: S1 Text — (PDF) [file pmed.1002933.s002.pdf]

S1 Text. Analysis plan

Association of blood pressure variability with the risk of dementia: a prospective cohort study

Background

Dementia is one of the most common progressive neurological diseases associated with disability and dependency in the elderly, and it is estimated that around 50 million individuals are living with dementia globally [1]. With the increase of life expectancy, the number of people living with dementia will continue to increase and is predicted to triple in 2050 [1,2].

High blood pressure is a major contributor not only to cardiovascular events but also to accelerated cognitive decline and dementia [2,3]. However, It has also been shown that the relationships between blood pressure level and health outcomes appear to attenuate with aging in late life [4,5]. Specifically, the association of elevated blood pressure with risk of dementia is controversial, with a positive association at mid-life and a likely inverse relationship in late life [6]. This inconsistency has impeded the potential of leveraging available antihypertensive treatment to prevent dementia. Emerging evidence, from post-hoc analysis of clinical trials and systematic review, suggests that blood pressure variability may relate to vascular-related outcomes, above and beyond the effect of mean blood pressure [7-9]. Indeed, several studies have reported that higher blood pressure variability was associated with impaired cognitive function independent of mean blood pressure in both young adults [10] and the aging population [11-13]. A few cohort studies also reported that both higher visit-to-visit and day-to-day BPV were associated with a higher risk of dementia, but the temporal order of the association was not fully clarified due to the short follow-ups of most studies [14-16]. The underlying mechanisms also remain poorly understood. We hypothesize that large blood pressure variability is associated with a higher risk of dementia, and that arterial stiffness may modify this putative association.

Analysis Objective

To investigate whether blood pressure variability is associated with the diagnosis of incident dementia, and to further examine the potential mechanisms, specifically the role of arterial remodeling in a prospective cohort study.

Study Design and Participants

This study will be conducted in The Rotterdam Study using a prospective cohort study design. Subjects without dementia during baseline measurements are eligible for the proposed study. A total of ~6000 participants from the original cohort (RS-I) initiated in year 1989 will be included.

Outcome-Assessment of Incident Dementia

- **The primary outcome** is all-cause incident dementia during the follow-up period.
- **The secondary outcomes** are major subtypes of dementia, i.e. Alzheimer's disease and vascular dementia.

Exposure-Blood Pressure Variability (BPV)

Visit-to-visit blood pressure variability will be calculated considering the following aspects:

- Given that blood pressure was measured every 3-4 years with relatively long and differential visit intervals, methods accounting for visit intervals will be considered.
- The direction of blood pressure variability will be considered, as the variation over 3-4 years could be profound, reflecting either a decreasing or an increasing trend.
- The calculation of BPV will account for average blood pressure level.
- Given the visit intervals of 3-4 years on average, which is significantly longer than previous studies on visit-to-visit BP variability and health outcomes. Measures of BPV, such as coefficient of variation, that require BP measured from three or more visits may not be suitable for the current study, as it will introduce issues associated with the assessment of sustained exposure, such as immortal time bias. Coefficient of variation (CV), standard deviation (SD) and average successive variability (ASV) will therefore be used as secondary measures to allow for the comparison with previous studies.

Other Measurements

Covariates for potential confounding control include:

(1) **time invariant covariates:** age, sex, education, and ApoE genotype;

(2) **time-varying covariates:** lifestyle factors (smoking, alcohol drinking), total cholesterol level, high-density lipoprotein, mean body weight and mean blood pressure, antihypertensive drug treatment, and history of diabetes and CVD.

Potential effect modifier: indicators of arterial stiffness **(**assessed by carotid-femoral pulse wave velocity index).

Statistical Methods

Primary Analysis

Primary Research Question: Is BPV associated with the risk of incident dementia?
The association of BPV with dementia will be examined using Cox proportional-hazard models with adjustment of aforementioned covariates considering the following aspects.

- Informative censoring will be accounted for using inverse probability weighting.
- BPV will be evaluated first as time-dependent variable, and then as time-independent variable (with first three waves as baseline measurement).
- In this analysis, any dementia cases reported during varied measurement period (with lag periods ranging from 0 years to the median of follow-up period) will be censored to assess potential reverse causation.

- BPV will be first entered as continuous and then as categorical (based on quantile cut-off points).
- To further allow for nonlinearity, smoothing splines will be employed.
- The above analysis will be repeated for vascular dementia and Alzheimer's disease.
- The above analysis will also be repeated for variability in DBP and pulse pressure.

Secondary Analysis

(1) To test whether arterial stiffness modify the association between BPV and the risk of incident dementia, analysis stratified by pulse wave velocity index will be performed. The interaction between arterial stiffness and BPV will be examined by adding a product term into the model.
(2) Pre-specified subgroup analyses: by age, sex, hypertension status, baseline blood pressure level and antihypertensive drug treatment;
(3) Correlation between different types of BPV and BP level.

Sensitivity Analysis

Several sensitivity analyses will be performed, including changing the eligibility criteria of study population, using different methods to calculate blood pressure variability, and testing different model assumptions.

Missing Value Handling

Missing values will be handled using the missing indicator procedure if the proportion of missing data is small (<20%), otherwise more complex approaches, such as multiple imputation or model-based methods, will be used where appropriate.

Software

All the analyses will be performed using SAS and R.

References

1. World Health Organization. Dementia fact sheet. 2017.
2. Livingston G, Sommerlad A, Orgeta V, Costafreda SG, Huntley J, Ames D, et al. Dementia prevention, intervention, and care. The Lancet. 2017. doi: 10.1016/S0140-6736(17)31363-6.
3. Lawes CM, Vander Hoorn S, Rodgers A. Global burden of blood-pressure-related disease, 2001. Lancet. 2008;371(9623):1513-8. doi: 10.1016/s0140-6736(08)60655-8.
4. Pickering TG, Hall JE, Appel LJ, Falkner BE, Graves J, Hill MN, et al. Recommendations for blood pressure measurement in humans and experimental animals: part 1: blood pressure measurement in humans: a statement for professionals from the Subcommittee of Professional and Public Education of the American Heart Association Council on High Blood Pressure Research. Circulation. 2005;111(5):697-716. doi: 10.1161/01.cir.0000154900.76284.f6.
5. Lewington S, Clarke R, Qizilbash N, Peto R, Collins R. Age-specific relevance of usual blood pressure to vascular mortality: a meta-analysis of individual data for one million adults in 61 prospective studies. Lancet. 2002;360(9349):1903-13.
6. Walker KA, Power MC, Gottesman RF. Defining the Relationship Between Hypertension, Cognitive Decline, and Dementia: a Review. Current hypertension reports. 2017;19(3):24. doi: 10.1007/s11906-017-0724-3.
7. Rothwell PM. Limitations of the usual blood-pressure hypothesis and importance of variability, instability, and episodic hypertension. Lancet. 2010;375(9718):938-48. doi: 10.1016/s0140-6736(10)60309-1.
8. Rothwell PM, Howard SC, Dolan E, O'Brien E, Dobson JE, Dahlof B, et al. Prognostic significance of visit-to-visit variability, maximum systolic blood pressure, and episodic hypertension. Lancet. 2010;375(9718):895-905. doi: 10.1016/s0140-6736(10)60308-x.
9. Stevens SL, Wood S, Koshiaris C, Law K, Glasziou P, Stevens RJ, et al. Blood pressure variability and cardiovascular disease: systematic review and meta-analysis. BMJ. 2016;354:i4098. doi: 10.1136/bmj.i4098.
10. Yano Y, Ning H, Allen N, Reis JP, Launer LJ, Liu K, et al. Long-term blood pressure variability throughout young adulthood and cognitive function in midlife: the Coronary Artery Risk Development in Young Adults (CARDIA) study. Hypertension. 2014;64(5):983-8. doi: 10.1161/hypertensionaha.114.03978.
11. Sabayan B, Wijsman LW, Foster-Dingley JC, Stott DJ, Ford I, Buckley BM, et al. Association of visit-to-visit variability in blood pressure with cognitive function in old age: prospective cohort study. BMJ. 2013;347:f4600. doi: 10.1136/bmj.f4600.
12. Wijsman LW, de Craen AJ, Muller M, Sabayan B, Stott D, Ford I, et al. Blood Pressure Lowering Medication, Visit-to-Visit Blood Pressure Variability, and Cognitive Function in Old Age. Am J Hypertens. 2016;29(3):311-8. doi: 10.1093/ajh/hpv101.
13. McDonald C, Pearce MS, Kerr SR, Newton JL. Blood pressure variability and cognitive decline in older people: a 5-year longitudinal study. J Hypertens. 2017;35(1):140-7. doi: 10.1097/hjh.0000000000001120.
14. Tully PJ, Dartigues JF, Debette S, Helmer C, Artero S, Tzourio C. Dementia risk with antihypertensive use and blood pressure variability: A cohort study. Neurology. 2016;87(6):601-8. doi: 10.1212/wnl.0000000000002946.

15. Oishi E, Ohara T, Sakata S, Fukuhara M, Hata J, Yoshida D, et al. Day-to-Day Blood Pressure Variability and Risk of Dementia in a General Japanese Elderly Population: The Hisayama Study. Circulation. 2017;136(6):516-25.
16. Alperovitch A, Blachier M, Soumare A, Ritchie K, Dartigues JF, Richard-Harston S, et al. Blood pressure variability and risk of dementia in an elderly cohort, the Three-City Study. Alzheimers Dement. 2014;10(5 Suppl):S330-7. doi: 10.1016/j.jalz.2013.05.1777.
