# Supplementary material for: Variation in blood pressure and long-term risk of dementia: A population-based cohort study
Source: PLoS Med. 2019 Nov 12;16(11):e1002933. doi: 10.1371/journal.pmed.1002933 (PMC6850672; doi:10.1371/journal.pmed.1002933)
Supplement: S2 Text — (DOCX) [file pmed.1002933.s003.docx]

**S2 Text Supplementary tables and figures**

**Supplementary Tables**

- **Table A.** Baseline characteristics of the study population.
- **Table B.** SBP variation and the risk of dementia by baseline SBP level.
- **Table C.** SBP variation and the risk of dementia by age and sex.
- **Table D.** SBP variation and the risk of dementia by pulse wave velocity.
- **Table E.** SBP variation and the risk of major subtypes of dementia.
- **Table F.** DBP variation and the risk of dementia.
- **Table G.** Pulse pressure variation and the risk of dementia.
- **Table H.** SBP variation (in mmHg per year) and the risk of dementia.
- **Table I.** Cause-specific hazard ratios for dementia and mortality in relation to SBP variation.
- **Table J.** Other measures of SBP variation and the risk of dementia.
- **Table K.** Correlation between SBP level and SBP variation.
- **Table L.** Sensitivity analyses on SBP variation and the risk of dementia.

**Supplementary Figures**

- **Fig A**. Flowchart of the study population
- **Fig B**. Lag-specific association of SBP variation with the risk of dementia.

| **Table A. Baseline characteristics of the study population** | | | | |
| --- | --- | --- | --- | --- |
|  | **The original cohort (n=7983)** | **Eligible  (n=5273)** | **Ineligible  (n=2710)** | **P-values ^a^** |
| Age at entry, years | 70.7 ± 9.8 | 67.6 ± 8.0 | 76.7 ± 10.3 | <0.001 |
| Women (%) | 4878 (61.1) | 3063 (58.1) | 1815 (67.0) | <0.001 |
| Education, n (%) |  |  |  | <0.001 |
| *lower* | 2016 (26.37) | 1037 (19.9) | 979 (40.4) |  |
| *Intermediate* | 5000 (65.4) | 3698 (70.8) | 1302 (53.7) |  |
| *Higher* | 629 (8.23) | 486 (9.3) | 143 (5.9) |  |
| APOE genotype, n (%) |  |  |  | 0.36 |
| *ε3/ε3* | 3990 (58.2) | 2957 (58.4) | 1033 (57.6) |  |
| *ε2/ε2 or ε2/ε3* | 928 (13.5) | 696 (13.8) | 232 (12.9) |  |
| *ε2/ε4, ε3/ε4, or ε4/ε4* | 1935 (28.3) | 1407 (27.8) | 528 (29.5) |  |
| Systolic blood pressure, mmHg | 139±22 | 138 ± 22 | 143 ± 24 | <0.001 |
| Diastolic blood pressure, mmHg | 74±12 | 74 ± 11 | 73 ± 13 | 0.006 |
| Pulse pressure, mmHg | 66±18 | 64 ± 17 | 70 ± 20 | <0.001 |
| Hypertension, n (%) | 4738 (64.1) | 3100 (58.8) | 1638 (77.1) | <0.001 |
| Smoking status at baseline, n (%) |  |  |  | <0.001 |
| *Never* | 2906 (39.0) | 1845 (36.3) | 1061 (44.7) |  |
| *Past* | 2994 (40.1) | 2184 (42.9) | 810 (34.1) |  |
| *Current* | 1559 (20.9) | 1057 (20.8) | 502 (21.2) |  |
| Current alcohol drinking, n (%) | 4305 (79.2) | 3621 (80.8) | 684 (71.8) | <0.001 |
| Body mass index, kg/m^2^ | 26.3 ± 3.7 | 26.3 ± 3.6 | 26.1 ± 4.0 | 0.009 |
| Total cholesterol, mmol/L | 6.6 ± 1.2 | 6.7 ± 1.2 | 6.4 ± 1.3 | <0.001 |
| HDL cholesterol, mmol/L | 1.3 ± 0.4 | 1.4 ± 0.4 | 1.3 ± 0.4 | 0.02 |
| MMSE score, points | 27 ± 3 | 28 ± 2 | 25 ± 5 | <0.001 |
| Diabetes, n (%) | 596 (8.9) | 336 (6.7) | 260 (15.1) | <0.001 |
| Stroke, n (%) | 261 (3.3) | 101 (1.9) | 160 (5.9) | <0.001 |
| Coronary heart disease, n (%) | 610 (8.0) | 373 (7.1) | 237 (10.0) | <0.001 |
| Atrial fibrillation, n (%) | 378 (5.6) | 203 (3.8) | 175 (10.5) | <0.001 |
| Heart failure, n (%) | 257 (3.3) | 121 (2.3) | 136 (5.5) | <0.001 |
| ^a^ A comparison of eligible versus ineligible subjects. Data are shown in the format of mean ± SD and n (%) unless otherwise specified. MMSE=Mini-Mental State Examination. | | | | |

| **Table B. SBP variation and the risk of dementia by baseline SBP level** | | | | | | | |
| --- | --- | --- | --- | --- | --- | --- | --- |
| Lag periods | Events/ | Hazard ratios (95%CI) of all-cause dementia ^a^ | | | | | |
|  | Participants at risk | Quintile 1  (<1.0%/year) | Quintile 2  (1.0~2.1%/year) | Quintile 3  (2.1~3.6%/year) | Quintile 4  (3.6~5.9%/year) | Quintile 5  (≥5.9%/year) | *P for linear trend* |
| Hazard ratios by systolic blood pressure at cohort entry ^b^ | | | | | | | |
| **<140mmHg** | | | | | | | |
| Lag 0 | 555/2905 | 1 (ref) | 1.09 (0.85 - 1.40) | 1.01 (0.78 - 1.30) | 0.92 (0.70 - 1.20) | 0.94 (0.71 - 1.26) | 0.375 |
| Lag 5 | 486/2613 | 1 (ref) | 1.05 (0.79 - 1.38) | 1.16 (0.88 - 1.53) | 1.17 (0.88 - 1.56) | 1.82 (1.34 - 2.45) | <.001 |
| Lag 10 | 339/2089 | 1 (ref) | 0.92 (0.63 - 1.36) | 1.19 (0.82 - 1.73) | 1.08 (0.73 - 1.59) | 1.86 (1.21 - 2.85) | 0.011 |
| Lag 15 | 217/1637 | 1 (ref) | 1.18 (0.65 - 2.12) | 1.33 (0.76 - 2.33) | 1.38 (0.77 - 2.49) | 5.27 (3.05 - 9.12) | <.001 |
| **≥140 mmHg** | | | | | | | |
| Lag 0 | 504/2368 | 1 (ref) | 1.11 (0.82 - 1.48) | 1.20 (0.90 - 1.61) | 1.43 (1.08 - 1.90) | 1.31 (0.95 - 1.80) | 0.016 |
| Lag 5 | 392/1919 | 1 (ref) | 1.34 (0.97 - 1.84) | 1.11 (0.80 - 1.56) | 1.63 (1.20 - 2.22) | 2.40 (1.69 - 3.43) | <.001 |
| Lag 10 | 247/1383 | 1 (ref) | 1.18 (0.79 - 1.78) | 0.93 (0.58 - 1.50) | 1.23 (0.83 - 1.80) | 1.41 (0.86 - 2.30) | 0.206 |
| Lag 15 | 143/928 | 1 (ref) | 0.85 (0.40 - 1.78) | 1.18 (0.52 - 2.68) | 2.12 (1.03 - 4.37) | 1.68 (0.83 - 3.43) | 0.013 |
| ^a^ Covariates adjustment was consistent with the final model in the primary analysis. | | | | | | | |
| ^b^ P values for interaction for baseline systolic blood pressure (with systolic blood pressure variation) were 0.22 (lag0), 0.51 (lag5), 0.18 (lag10), and 0.001 (lag15). | | | | | | | |

| **Table C. SBP variation and the risk of dementia by age and sex** | | | | | | | |
| --- | --- | --- | --- | --- | --- | --- | --- |
| Lag periods | Events/ | Hazard ratios (95%CI) of all-cause dementia ^a^ | | | | | |
|  | Participants at risk | Quintile 1  (<1.0%/year) | Quintile 2  (1.0~2.1%/year) | Quintile 3  (2.1~3.6%/year) | Quintile 4  (3.6~5.9%/year) | Quintile 5  (≥5.9%/year) | *P for linear trend* |
| Hazard ratios by age group ^b^ | | | | | | | |
| **< 70 years** | | | | | | | |
| Lag 0 | 265/2240 | 1 (ref) | 1.01 (0.78 - 1.30) | 1.03 (0.80 - 1.33) | 1.16 (0.89 - 1.51) | 1.00 (0.72 - 1.40) | 0.517 |
| Lag 5 | 255/2137 | 1 (ref) | 1.25 (0.96 - 1.64) | 1.35 (1.03 - 1.76) | 1.58 (1.2 - 2.09) | 2.42 (1.77 - 3.31) | <0.001 |
| Lag 10 | 215/1918 | 1 (ref) | 1.03 (0.74 - 1.43) | 1.23 (0.9 - 1.69) | 1.21 (0.87 - 1.66) | 2.11 (1.45 - 3.06) | <0.001 |
| Lag 15 | 165/1668 | 1 (ref) | 1.02 (0.65 - 1.62) | 1.44 (0.92 - 2.25) | 1.76 (1.14 - 2.72) | 4.03 (2.70 - 6.03) | <0.001 |
| **>= 70 years** | | | | | | | |
| Lag 0 | 794/3033 | 1 (ref) | 1.27 (0.95 - 1.69) | 1.24 (0.92 - 1.67) | 1.17 (0.88 - 1.55) | 1.22 (0.91 - 1.63) | 0.372 |
| Lag 5 | 623/2395 | 1 (ref) | 1.09 (0.78 - 1.52) | 0.96 (0.68 - 1.36) | 1.15 (0.84 - 1.58) | 1.72 (1.21 - 2.43) | 0.007 |
| Lag 10 | 371/1554 | 1 (ref) | 1.14 (0.67 - 1.94) | 0.83 (0.45 - 1.52) | 1.10 (0.69 - 1.75) | 1.22 (0.72 - 2.07) | 0.542 |
| Lag 15 | 195/897 | 1 (ref) | 1.07 (0.32 - 3.63) | 1.43 (0.37 - 5.53) | 1.77 (0.53 - 5.96) | 2.57 (0.88 - 7.53) | 0.018 |
| Hazard ratios by sex ^c^ | | | | | | | |
| **Men** | | | | | | | |
| Lag 0 | 347/2366 | 1 (ref) | 1.14 (0.83 - 1.58) | 1.05 (0.76 - 1.46) | 1.18 (0.85 - 1.64) | 0.94 (0.65 - 1.37) | 0.999 |
| Lag 5 | 271/1996 | 1 (ref) | 0.97 (0.67 - 1.41) | 1.11 (0.76 - 1.63) | 1.25 (0.85 - 1.83) | 1.80 (1.16 - 2.80) | 0.011 |
| Lag 10 | 185/1526 | 1 (ref) | 0.68 (0.42 - 1.12) | 0.69 (0.42 - 1.13) | 0.88 (0.55 - 1.43) | 1.12 (0.57 - 2.21) | 0.701 |
| Lag 15 | 125/1135 | 1 (ref) | 0.79 (0.36 - 1.73) | 1.13 (0.50 - 2.54) | 1.83 (0.90 - 3.73) | 4.76 (2.43 - 9.32) | <0.001 |
| **Women** | | | | | | | |
| Lag 0 | 712/2907 | 1 (ref) | 1.08 (0.85 - 1.36) | 1.14 (0.90 - 1.44) | 1.11 (0.87 - 1.41) | 1.17 (0.90 - 1.51) | 0.240 |
| Lag 5 | 607/2536 | 1 (ref) | 1.23 (0.96 - 1.59) | 1.21 (0.94 - 1.56) | 1.40 (1.09 - 1.80) | 2.19 (1.67 - 2.88) | <0.001 |
| Lag 10 | 401/1946 | 1 (ref) | 1.27 (0.91 - 1.77) | 1.33 (0.94 - 1.86) | 1.25 (0.91 - 1.72) | 1.98 (1.39 - 2.83) | 0.002 |
| Lag 15 | 235/1430 | 1 (ref) | 1.18 (0.66 - 2.08) | 1.36 (0.75 - 2.46) | 1.60 (0.91 - 2.81) | 2.49 (1.46 - 4.26) | <0.001 |
| ^a^ Covariates adjustment was consistent with the final model in the primary analysis. | | | | | | | |
| ^b^ P values for interaction for age (with systolic blood pressure variation) were 0.67 (lag0), 0.20 (lag5), 0.30 (lag10), and 0.64 (lag15). | | | | | | | |
| ^c^ P values for interaction for sex (with systolic blood pressure variation) were 0.93 (lag0), 0.89 (lag5), 0.19 (lag10), and 0.03 (lag15). | | | | | | | |

| **Table D. SBP variation and the risk of dementia by pulse wave velocity** | | | | | | | |
| --- | --- | --- | --- | --- | --- | --- | --- |
| Lag periods  (years) | Events/ | Hazard ratios (95%CI) ^a^ | | | | | |
|  | Participants at risk | Quintile 1^b^  (<1.0%/year) | Quintile 2  (1.0~2.1%/year) | Quintile 3  (2.1~3.6%/year) | Quintile 4  (3.6~5.9%/year) | Quintile 5  (≥5.9%/year) | *P for linear trend* |
| Pulse Wave Velocity Index <14 meters/second ^c^ | | | | | | | |
| 0 | 345/1987 | 1 | 1.07 (0.79 - 1.44) | 0.96 (0.70 - 1.31) | 1.08 (0.78 - 1.51) | 1.13 (0.70 - 1.82) | 0.679 |
| 5 | 328/1961 | 1 | 0.92 (0.67 - 1.26) | 0.96 (0.70 - 1.32) | 1.08 (0.78 - 1.49) | 2.59 (1.77 - 3.78) | 0.001 |
| 10 | 252/1698 | 1 | 0.94 (0.61 - 1.44) | 1.08 (0.72 - 1.60) | 1.36 (0.91 - 2.03) | 2.50 (1.61 - 3.90) | <0.001 |
| 15 | 169/1406 | 1 | 0.83 (0.50 - 1.38) | 1.20 (0.69 - 2.06) | 1.22 (0.71 - 2.07) | 1.90 (1.16 - 3.11) | 0.003 |
| Pulse Wave Velocity Index >=14 meters/second | | | | | | | |
| 0 | 257/1204 | 1 | 1.28 (0.88 - 1.86) | 1.02 (0.69 - 1.51) | 1.04 (0.69 - 1.59) | 0.99 (0.54 - 1.81) | 0.776 |
| 5 | 230/1165 | 1 | 1.83 (1.22 - 2.77) | 1.44 (0.94 - 2.23) | 1.88 (1.21 - 2.91) | 3.45 (2.09 - 5.71) | <0.001 |
| 10 | 145/883 | 1 | 1.27 (0.72 - 2.21) | 0.98 (0.52 - 1.86) | 1.30 (0.74 - 2.29) | 1.87 (0.93 - 3.76) | 0.133 |
| 15 | 81/580 | 1 | 1.92 (0.59 - 6.24) | 1.87 (0.51 - 6.89) | 3.30 (1.05 - 10.42) | 7.50 (2.4 - 23.39) | <0.001 |
| ^a^ Covariates adjustment was consistent with the final model in the primary analysis.  ^b^ Reference category.  ^c^ Dichotomized according to the upper tertile of continuous measurements on pulse wave velocity index.  P values for interaction for pulse wave velocity index (with systolic blood pressure variation) were 0.75 (lag0), 0.05 (lag5), 0.70 (lag10), and 0.65 (lag15). | | | | | | | |

| **Table E. SBP variation and the risk of major subtypes of dementia** | | | | | | | |
| --- | --- | --- | --- | --- | --- | --- | --- |
| Lag periods  (years) | Events/ | Hazard ratios (95%CI) ^a^ | | | | | |
|  | Participants at risk | Quintile 1^b^  (<1.0%/year) | Quintile 2  (1.0~2.1%/year) | Quintile 3  (2.1~3.6%/year) | Quintile 4  (3.6~5.9%/year) | Quintile 5  (≥5.9%/year) | *P for linear trend* |
| Hazard ratios of Alzheimer's disease | | | | | | | |
| 0 | 802/5273 | 1 | 1.07 (0.86 - 1.33) | 1.09 (0.88 - 1.36) | 1.02 (0.81 - 1.27) | 1.07 (0.84 - 1.37) | 0.743 |
| 5 | 667/4532 | 1 | 1.08 (0.85 - 1.37) | 1.02 (0.80 - 1.30) | 1.22 (0.96 - 1.55) | 1.96 (1.51 - 2.55) | <0.001 |
| 10 | 431/3472 | 1 | 1.02 (0.73 - 1.43) | 1.09 (0.78 - 1.53) | 1.25 (0.91 - 1.70) | 1.86 (1.30 - 2.66) | 0.002 |
| 15 | 255/2565 | 1 | 0.93 (0.52 - 1.65) | 1.12 (0.64 - 1.97) | 1.41 (0.84 - 2.37) | 3.01 (1.83 - 4.94) | <0.001 |
| Hazard ratios of vascular dementia ^c^ | | | | | | | |
| 0 | 80/5273 | 1 | 2.18 (0.94 - 5.05) | 1.81 (0.74 - 4.44) | 2.66 (1.13 - 6.26) | 2.17 (0.90 - 5.20) | 0.078 |
| 5 | 57/4532 | 1 | 1.78 (0.68 - 4.66) | 1.88 (0.72 - 4.88) | 1.59 (0.58 - 4.38) | 4.31 (1.66 - 11.21) | 0.011 |
| 10 | 36/3472 | 1 | 1.75 (0.50 - 6.17) | 3.27 (1.04 - 10.27) | 1.74 (0.50 - 6.08) | 4.50 (1.26 - 16.09) | 0.055 |
| ^a^ Covariates adjustment was consistent with the final model in the primary analysis.  ^b^ Reference category.  ^c^ Lag 15 analysis was not performed due to a small number of cases. | | | | | | | |

| **Table F. DBP variation and the risk of dementia** | | | | | | | |
| --- | --- | --- | --- | --- | --- | --- | --- |
| Lag periods | Events/ | Hazard ratios (95%CI) ^a^ | | | | | |
| (years) | Participants at risk | Quintile 1^b^ (<1.1%/year) | Quintile 2 (1.1~2.2%/year) | Quintile 3 (2.2~3.8%/year) | Quintile 4 (3.8~6.6%/year) | Quintile 5 (≥ 6.6%/year) | *P for linear trend* |
| 0 | 1059/5273 | 1 | 1.15 (0.96 - 1.39) | 1.03 (0.85 - 1.25) | 1.06 (0.87 - 1.29) | 1.03 (0.83 - 1.28) | 0.952 |
| 5 | 878/4532 | 1 | 1.05 (0.85 - 1.28) | 1.04 (0.84 - 1.28) | 1.13 (0.91 - 1.40) | 2.03 (1.61 - 2.55) | <0.001 |
| 10 | 586/3472 | 1 | 0.95 (0.71 - 1.28) | 1.18 (0.89 - 1.56) | 1.32 (1.00 - 1.75) | 1.94 (1.40 - 2.69) | <0.001 |
| 15 | 360/2565 | 1 | 0.98 (0.60 - 1.59) | 1.49 (0.96 - 2.31) | 2.02 (1.37 - 2.99) | 3.10 (2.06 - 4.68) | <0.001 |
| ^a^ With adjustment for age, sex, education level, *APOE* genotype and time-dependent covariates on smoking habit, alcohol consumption, the use of antihypertensive medication, body mass index, lipid level, and history of diabetes and cardiovascular disease.  ^b^ Reference category. | | | | | | | |

| **Table G. Pulse pressure variation and the risk of dementia** | | | | | | | |
| --- | --- | --- | --- | --- | --- | --- | --- |
| Lag periods | Events/ | Hazard ratios (95%CI) ^a^ | | | | | |
| (years) | Participants at risk | Quintile 1^b^ (<1.7%/year) | Quintile 2 (1.7~3.5%/year) | Quintile 3 (3.5~5.9%/year) | Quintile 4 (5.9~9.9%/year) | Quintile 5 (≥ 9.9%/year) | *P for linear trend* |
| 0 | 1059/5273 | 1 | 1.01 (0.84 - 1.21) | 0.88 (0.72 - 1.07) | 1.1 (0.91 - 1.33) | 1.07 (0.87 - 1.32) | 0.390 |
| 5 | 878/4532 | 1 | 1.02 (0.83 - 1.25) | 0.99 (0.79 - 1.22) | 1.4 (1.14 - 1.73) | 2.04 (1.62 - 2.56) | <0.001 |
| 10 | 586/3472 | 1 | 0.94 (0.72 - 1.23) | 1.01 (0.76 - 1.35) | 1.2 (0.91 - 1.58) | 1.66 (1.24 - 2.23) | <0.001 |
| 15 | 360/2565 | 1 | 0.99 (0.63 - 1.53) | 0.53 (0.34 - 0.83) | 1.11 (0.75 - 1.64) | 2.06 (1.41 - 3.01) | <0.001 |
| ^a^ With adjustment for age, sex, education level, *APOE* genotype and time-dependent covariates on smoking habit, alcohol consumption, the use of antihypertensive medication, body mass index, lipid level, and history of diabetes and cardiovascular disease.  ^b^ Reference category. | | | | | | | |

|  | | | | | | | |
| --- | --- | --- | --- | --- | --- | --- | --- |
| **Table H.** **SBP variation (in mmHg per year) and the risk of dementia** | | | | | | | |
| Lag periods  (Years) | Events/ | Hazard ratios (95%CI) ^a^ | | | | | |
|  | Participants at risk | Quintile 1^b^  (≤1.4 mmHg/year) | Quintile 2  (1.4~3.0 mmHg/year) | Quintile 3  (3.0~5.1 mmHg/year) | Quintile 4  (5.1~8.5mmHg/year) | Quintile 5  (≥ 8.5mmHg/year) | *P for trend* |
| Hazard ratios of all-cause dementia | | | | | | | |
| Lag 0 | 1059/5273 | 1 | 0.97 (0.76 - 1.24) | 1.06 (0.83 - 1.35) | 1.22 (0.96 - 1.55) | 1.02 (0.76 - 1.35) | 0.267 |
| Lag 5 | 878/4532 | 1 | 0.99 (0.78 - 1.25) | 1.23 (0.98 - 1.53) | 1.34 (1.06 - 1.69) | 2.24 (1.73 - 2.91) | <0.001 |
| Lag 10 | 586/3472 | 1 | 1.03 (0.76 - 1.38) | 1.21 (0.89 - 1.64) | 1.12 (0.83 - 1.52) | 1.90 (1.34 - 2.70) | 0.002 |
| Lag 15 | 360/2565 | 1 | 1.28 (0.83 - 1.99) | 1.04 (0.63 - 1.72) | 1.80 (1.180 - 2.76) | 3.53 (2.31 - 5.39) | <0.001 |
| ^a^ Hazard ratios after adjusting for age, sex, education level, APOE genotype and time-dependent covariates on smoking habit, alcohol consumption, the use of antihypertensive medication, body mass index, lipid level, and history of diabetes and cardiovascular disease.  ^b^ Reference level. | | | | | | | |

| **Table I. Cause-specific hazard ratios for dementia and mortality in relation to SBP variation** | | | | | | | |
| --- | --- | --- | --- | --- | --- | --- | --- |
| Lag periods  (years) | Events/ | Hazard ratios (95%CI) ^a^ | | | | | |
|  | Participants at risk | Quintile 1^b^  (<1.0%/year) | Quintile 2  (1.0~2.1%/year) | Quintile 3  (2.1~3.6%/year) | Quintile 4  (3.6~5.9%/year) | Quintile 5  (≥5.9%/year) | *P for linear trend* |
| Cause-specific hazard ratios for all-cause dementia | | | | | | | |
| 0 | 1059/5273 | 1 | 1.08 (0.90 - 1.31) | 1.09 (0.90 - 1.32) | 1.13 (0.93 - 1.37) | 1.08 (0.88 - 1.34) | 0.343 |
| 5 | 878/4532 | 1 | 1.12 (0.91 - 1.39) | 1.15 (0.93 - 1.42) | 1.33 (1.07 - 1.64) | 2.02 (1.61 - 2.53) | <0.001 |
| 10 | 586/3472 | 1 | 1.05 (0.80 - 1.37) | 1.11 (0.85 - 1.44) | 1.15 (0.89 - 1.49) | 1.66 (1.26 - 2.20) | 0.001 |
| 15 | 360/2565 | 1 | 1.04 (0.68 - 1.59) | 1.32 (0.87 - 1.98) | 1.76 (1.20 - 2.60) | 3.13 (2.17 - 4.52) | <0.001 |
| Cause-specific hazard ratios for all-cause mortality | | | | | | | |
| 0 | 2805/5273 | 1 | 1.00 (0.89 - 1.13) | 1.00 (0.89 - 1.12) | 0.94 (0.83 - 1.06) | 1.07 (0.95 - 1.22) | 0.717 |
| 5 | 2256/4532 | 1 | 0.99 (0.87 - 1.13) | 1.04 (0.91 - 1.19) | 1.12 (0.98 - 1.27) | 2.03 (1.78 - 2.32) | <0.001 |
| 10 | 1518/3472 | 1 | 0.91 (0.77 - 1.07) | 0.89 (0.76 - 1.05) | 1.09 (0.94 - 1.27) | 1.64 (1.40 - 1.92) | <0.001 |
| 15 | 853/2565 | 1 | 1.27 (1.02 - 1.58) | 1.03 (0.82 - 1.30) | 1.40 (1.13 - 1.74) | 2.07 (1.68 - 2.57) | <0.001 |
| ^a^ Covariates adjustment was consistent with the final model in the primary analysis.  ^b^ Reference category. | | | | | | | |

| **Table J. Other measures of SBP variation and the risk of dementia** | | | |
| --- | --- | --- | --- |
| Measures on BP variation ^a^ | Events/ Participants at risk | Level of BP variation ^e^ | Hazard ratios (95%CI) of all-cause dementia ^f^ |
| COV (%) ^b^ | | | |
| Quintile 1 | 126/749 | 3.0 (< 4.6) | 1 (ref) |
| Quintile 2 | 140/750 | 5.9 [4.6, 7.1) | 1.11 (0.87 - 1.41) |
| Quintile 3 | 137/750 | 8.4 [7.1, 9.7) | 1.01 (0.79 - 1.29) |
| Quintile 4 | 146/750 | 11.0 [ 9.7, 12.8) | 1.15 (0.90 - 1.47) |
| Quintile 5 | 152/750 | 15.7 (>= 12.8) | 1.29 (1.01 - 1.66) |
| *P* for trend | - | - | 0.055 |
| ASV (mmHg) ^c^ | | | |
| Quintile 1 | 122/742 | 5.0 (< 7.5) | 1 (ref) |
| Quintile 2 | 131/711 | 9.5 [7.5, 11.5) | 1.14 (0.89 - 1.46) |
| Quintile 3 | 147/806 | 13.5 [11.5, 16.5) | 1.05 (0.83 - 1.35) |
| Quintile 4 | 143/723 | 19.0 [16.3, 22.5) | 1.15 (0.90 - 1.48) |
| Quintile 5 | 158/767 | 27.8 (>= 22.5) | 1.26 (0.98 - 1.61) |
| *P* for trend | - | - | 0.087 |
| SD (mmHg) ^d^ | | | |
| Quintile 1 | 126/754 | 4.2 (< 6.3) | 1 (ref) |
| Quintile 2 | 133/747 | 8.1 [6.3, 9.8) | 0.96 (0.75 - 1.23) |
| Quintile 3 | 137/751 | 11.5 [9.8, 13.3) | 1.01 (0.79 - 1.30) |
| Quintile 4 | 146/746 | 15.3 [13.3, 18.0) | 1.09 (0.86 - 1.40) |
| Quintile 5 | 159/751 | 22.5 (>= 18.0) | 1.22 (0.95 - 1.57) |
| *P* for trend | - | - | 0.060 |
| ^a^ All measures were Derived from BP measured at the first three visits. ^b^ COV=coefficient of variation; COV was calculated as standard deviation divided by mean BP. ^c^ ASV=average successive variability; ASV was calculated as the average absolute difference between consecutive BP measurements. ^d^ SD=standard deviation.  ^e^ Median (range) ^f^ Hazard ratios were estimated from Cox proportional hazard ratio models, after accounting for age, sex, education level, APOE genotype, baseline covariates including smoking, alcohol drinking, body mass index, lipid level, history of diabetes and CVD, and mean systolic BP of the first three visits. | | | |

| **Table K. Correlation between SBP level and SBP variation** ^a^ | | | | | | |
| --- | --- | --- | --- | --- | --- | --- |
|  | mean SBP at baseline | mean SBP during two sequential visits | Variation in SBP across sequential visits ^b^ | Coefficient of variation ^c^ | Standard deviation ^c^ | Average real variability ^c, d^ |
| mean baseline SBP | 1.00 | 0.66 | 0.01 | -0.14 | 0.02 | 0.07 |
| mean SBP between sequential visits |  | 1.00 | -0.05 | 0.03 | 0.19 | 0.18 |
| Variation in SBP between sequential visits |  |  | 1.00 | 0.41 | 0.42 | 0.48 |
| Coefficient of variation |  |  |  | 1.00 | 0.98 | 0.92 |
| Standard deviation |  |  |  |  | 1 | 0.94 |
| Average real variability |  |  |  |  |  | 1 |
| ^a^ Spearman's correlation coefficients. All p values<0.01 except the correlation between mean SBP at baseline and variability measured by coefficient of variation (p=0.09).  ^b^ primary measure of BP variation assessed by BP between sequential visits as a time-varying exposure.  ^c^ derived from BP measured from the first three visits spanning six years.  ^d^ calculated as the average absolute difference between consecutive BP measurements. | | | | | | |

| **Table L. Sensitivity analyses on SBP variation and the risk of dementia** | | | | | | | |
| --- | --- | --- | --- | --- | --- | --- | --- |
|  | Events | Hazard ratios (95% CI) ^a^ | | | | | |
|  | /Participants at risk | Quintile 1^b^ | Quintile 2 | Quintile 3 | Quintile 4 | Quintile 5 | *P for linear trend* |
|  |  | (<1.0%/year) | (1.0~2.1%/year) | (2.1~3.6%/year) | (3.6~5.9%/year) | (≥5.9%/year) |  |
| **Excluding participants with prevalent cardiovascular disease at baseline** | | | | | | | |
| Lag0 | 924/4604 | 1 | 1.08 (0.89 - 1.32) | 1.07 (0.87 - 1.31) | 1.12 (0.92 - 1.38) | 1.08 (0.86 - 1.36) | 0.400 |
| Lag5 | 789/4056 | 1 | 1.18 (0.95 - 1.47) | 1.19 (0.96 - 1.49) | 1.39 (1.12 - 1.73) | 1.97 (1.54 - 2.52) | <0.001 |
| Lag10 | 543/3198 | 1 | 1.13 (0.84 - 1.52) | 1.15 (0.85 - 1.56) | 1.25 (0.95 - 1.66) | 2.00 (1.46 - 2.75) | <0.001 |
| Lag15 | 341/2415 | 1 | 1.13 (0.69 - 1.86) | 1.25 (0.75 - 2.07) | 1.73 (1.08 - 2.79) | 3.48 (2.24 - 5.39) | <0.001 |
| **Excluding participants with prevalent diabetes mellitus at baseline** | | | | | | | |
| Lag0 | 931/4655 | 1 | 1.08 (0.88 - 1.32) | 1.04 (0.84 - 1.27) | 1.13 (0.92 - 1.38) | 1.01 (0.80 - 1.26) | 0.698 |
| Lag5 | 776/4058 | 1 | 1.19 (0.95 - 1.48) | 1.14 (0.91 - 1.42) | 1.37 (1.10 - 1.71) | 2.10 (1.65 - 2.67) | <0.001 |
| Lag10 | 530/3166 | 1 | 0.96 (0.71 - 1.30) | 1.05 (0.77 - 1.43) | 1.15 (0.86 - 1.55) | 1.72 (1.22 - 2.41) | 0.003 |
| Lag15 | 326/2370 | 1 | 0.89 (0.54 - 1.47) | 1.38 (0.84 - 2.27) | 1.76 (1.10 - 2.82) | 3.40 (2.21 - 5.25) | <0.001 |
| **Censoring blood pressure measurements after the onset of cardiovascular disease** | | | | | | | |
| Lag0 | 900/4444 | 1 | 1.06 (0.86 - 1.30) | 1.10 (0.90 - 1.36) | 1.17 (0.95 - 1.44) | 1.07 (0.85 - 1.35) | 0.277 |
| Lag5 | 775/3945 | 1 | 1.11 (0.89 - 1.39) | 1.24 (0.99 - 1.55) | 1.52 (1.23 - 1.89) | 1.88 (1.46 - 2.41) | <0.001 |
| Lag10 | 534/3125 | 1 | 1.06 (0.78 - 1.42) | 1.07 (0.79 - 1.44) | 1.28 (0.97 - 1.69) | 1.95 (1.42 - 2.69) | <0.001 |
| Lag15 | 335/2369 | 1 | 1.14 (0.70 - 1.88) | 1.23 (0.76 - 1.99) | 1.97 (1.24 - 3.14) | 3.37 (2.18 - 5.22) | <0.001 |
| **Restricting analyses to individuals competing all planned visits before censoring** | | | | | | | |
| Lag0 | 1003/5088 | 1 | 1.10 (0.91 - 1.34) | 1.10 (0.91 - 1.34) | 1.12 (0.92 - 1.36) | 1.08 (0.87 - 1.34) | 0.405 |
| Lag5 | 846/4348 | 1 | 1.15 (0.93 - 1.42) | 1.15 (0.93 - 1.42) | 1.30 (1.05 - 1.62) | 2.09 (1.64 - 2.65) | <0.001 |
| Lag10 | 556/3292 | 1 | 1.06 (0.79 - 1.41) | 1.11 (0.82 - 1.50) | 1.13 (0.85 - 1.50) | 1.68 (1.20 - 2.35) | 0.012 |
| Lag15 | 342/2420 | 1 | 1.05 (0.65 - 1.70) | 1.30 (0.80 - 2.13) | 1.70 (1.07 - 2.70) | 3.31 (2.16 - 5.07) | <0.001 |
| ^a^ Covariates adjustment was consistent with the final model in the primary analysis.  ^b^ Reference category. | | | | | | | |

| **Table L. Sensitivity analyses on SBP variation and the risk of dementia (continued)** | | | | | | | |
| --- | --- | --- | --- | --- | --- | --- | --- |
|  | Events | Hazard ratios (95% CI) ^a^ | | | | | |
|  | /Participants at risk | Quintile 1^b^ | Quintile 2 | Quintile 3 | Quintile 4 | Quintile 5 | *P for linear trend* |
|  |  | (<1.0%/year) | (1.0~2.1%/year) | (2.1~3.6%/year) | (3.6~5.9%/year) | (≥5.9%/year) |  |
| **Estimates without using Inverse Probability Weighting** | | | | | | | |
| Lag0 | 1059/5273 | 1 | 1.08 (0.89 - 1.30) | 1.09 (0.90 - 1.32) | 1.12 (0.92 - 1.35) | 1.08 (0.87 - 1.34) | 0.374 |
| Lag5 | 878/4532 | 1 | 1.15 (0.93 - 1.42) | 1.18 (0.95 - 1.46) | 1.36 (1.10 - 1.68) | 2.08 (1.66 - 2.61) | <0.001 |
| Lag10 | 586/3472 | 1 | 1.02 (0.78 - 1.34) | 1.08 (0.83 - 1.41) | 1.19 (0.92 - 1.54) | 1.91 (1.44 - 2.53) | <0.001 |
| Lag15 | 360/2565 | 1 | 1.00 (0.67 - 1.51) | 1.21 (0.82 - 1.80) | 1.66 (1.14 - 2.42) | 3.36 (2.35 - 4.79) | <0.001 |
| **Imputing missing data using multiple imputation** | | | | | | | |
| Lag0 | 1059/5273 | 1 | 1.08 (0.90 - 1.31) | 1.09 (0.90 - 1.32) | 1.16 (0.96 - 1.40) | 1.15 (0.94 - 1.42) | 0.118 |
| Lag5 | 878/4532 | 1 | 1.12 (0.91 - 1.39) | 1.16 (0.94 - 1.43) | 1.24 (1.00 - 1.53) | 1.77 (1.42 - 2.21) | <0.001 |
| Lag10 | 586/3472 | 1 | 1.03 (0.79 - 1.35) | 1.10 (0.84 - 1.43) | 1.15 (0.89 - 1.49) | 1.66 (1.26 - 2.19) | 0.001 |
| Lag15 | 360/2565 | 1 | 1.03 (0.68 - 1.55) | 1.26 (0.85 - 1.86) | 1.61 (1.11 - 2.33) | 2.99 (2.1 - 4.25) | <0.001 |
| **Censoring participants at the diagnosis of stroke** | | | | | | | |
| Lag0 | 931/5273 | 1 | 1.02 (0.83 - 1.25) | 1.08 (0.88 - 1.32) | 1.14 (0.94 - 1.40) | 1.03 (0.82 - 1.30) | 0.373 |
| Lag5 | 762/4532 | 1 | 1.03 (0.82 - 1.30) | 1.19 (0.95 - 1.49) | 1.46 (1.17 - 1.82) | 1.95 (1.52 - 2.51) | <0.001 |
| Lag10 | 495/3472 | 1 | 0.98 (0.71 - 1.36) | 1.21 (0.88 - 1.65) | 1.11 (0.82 - 1.50) | 1.57 (1.11 - 2.24) | 0.017 |
| Lag15 | 299/2565 | 1 | 1.19 (0.70 - 2.03) | 1.31 (0.78 - 2.22) | 1.80 (1.08 - 3.01) | 3.19 (1.98 - 5.14) | <0.001 |
| **Additional adjustment for baseline cognitive function assessed by Mini-Mental State Examination score** | | | | | | | |
| Lag0 | 1059/5273 | 1 | 1.09 (0.90 - 1.32) | 1.09 (0.9 - 1.32) | 1.11 (0.92 - 1.35) | 1.08 (0.88 - 1.34) | 0.396 |
| Lag5 | 878/4532 | 1 | 1.12 (0.91 - 1.38) | 1.14 (0.93 - 1.41) | 1.31 (1.06 - 1.61) | 2.01 (1.60 - 2.54) | <0.001 |
| Lag10 | 586/3472 | 1 | 1.05 (0.79 - 1.40) | 1.10 (0.82 - 1.48) | 1.14 (0.87 - 1.51) | 1.67 (1.21 - 2.31) | 0.005 |
| Lag15 | 360/2565 | 1 | 1.05 (0.65 - 1.70) | 1.28 (0.79 - 2.07) | 1.70 (1.08 - 2.68) | 3.14 (2.06 - 4.78) | <0.001 |
| ^a^ Covariates adjustment was consistent with the final model in the primary analysis.  ^b^ Reference category. | | | | | | | |

**Fig A. Flowchart of the study population**


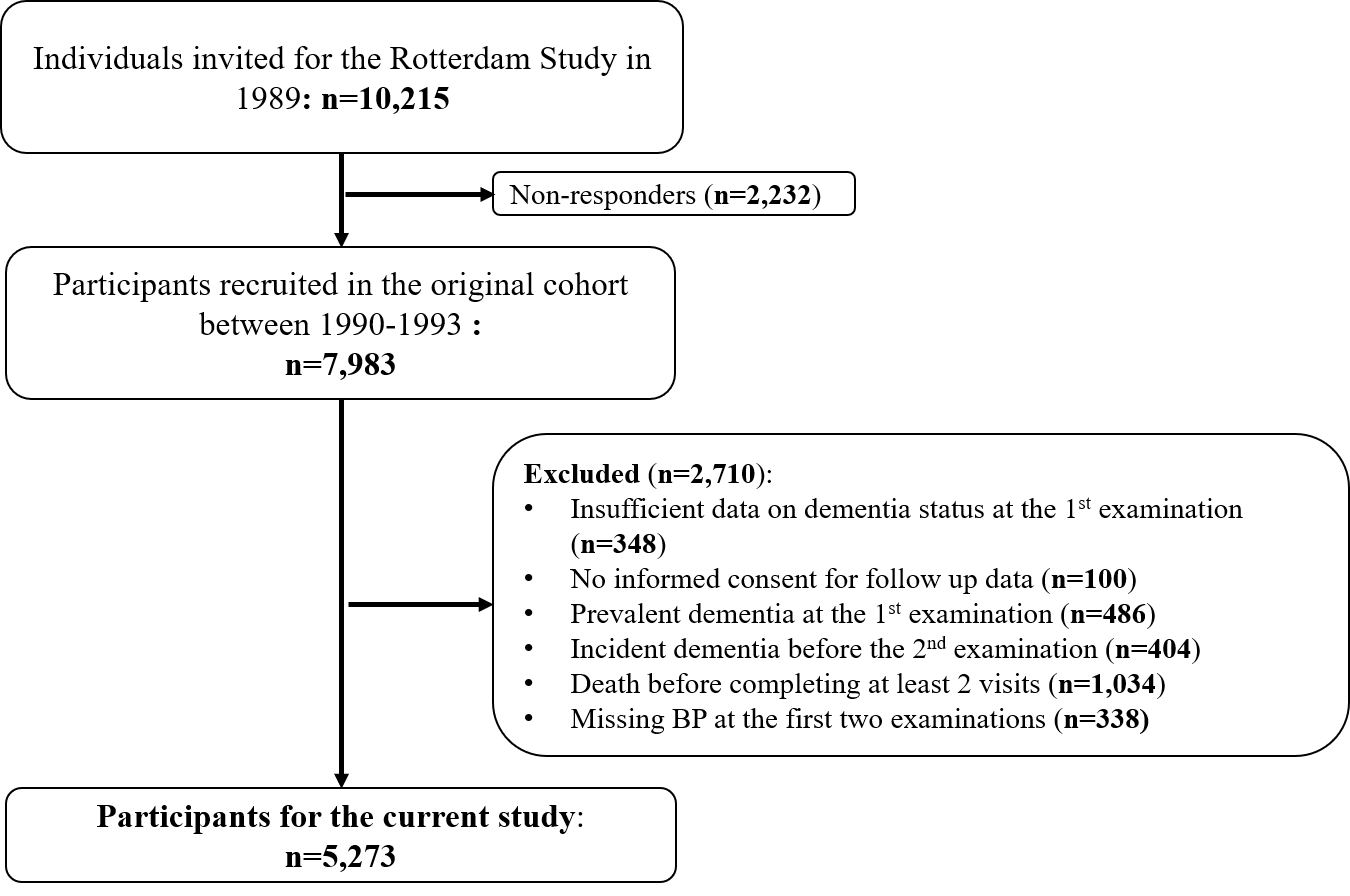


**Fig B. Lag-specific association of magnitude of SBP variation with the risk of dementia**.


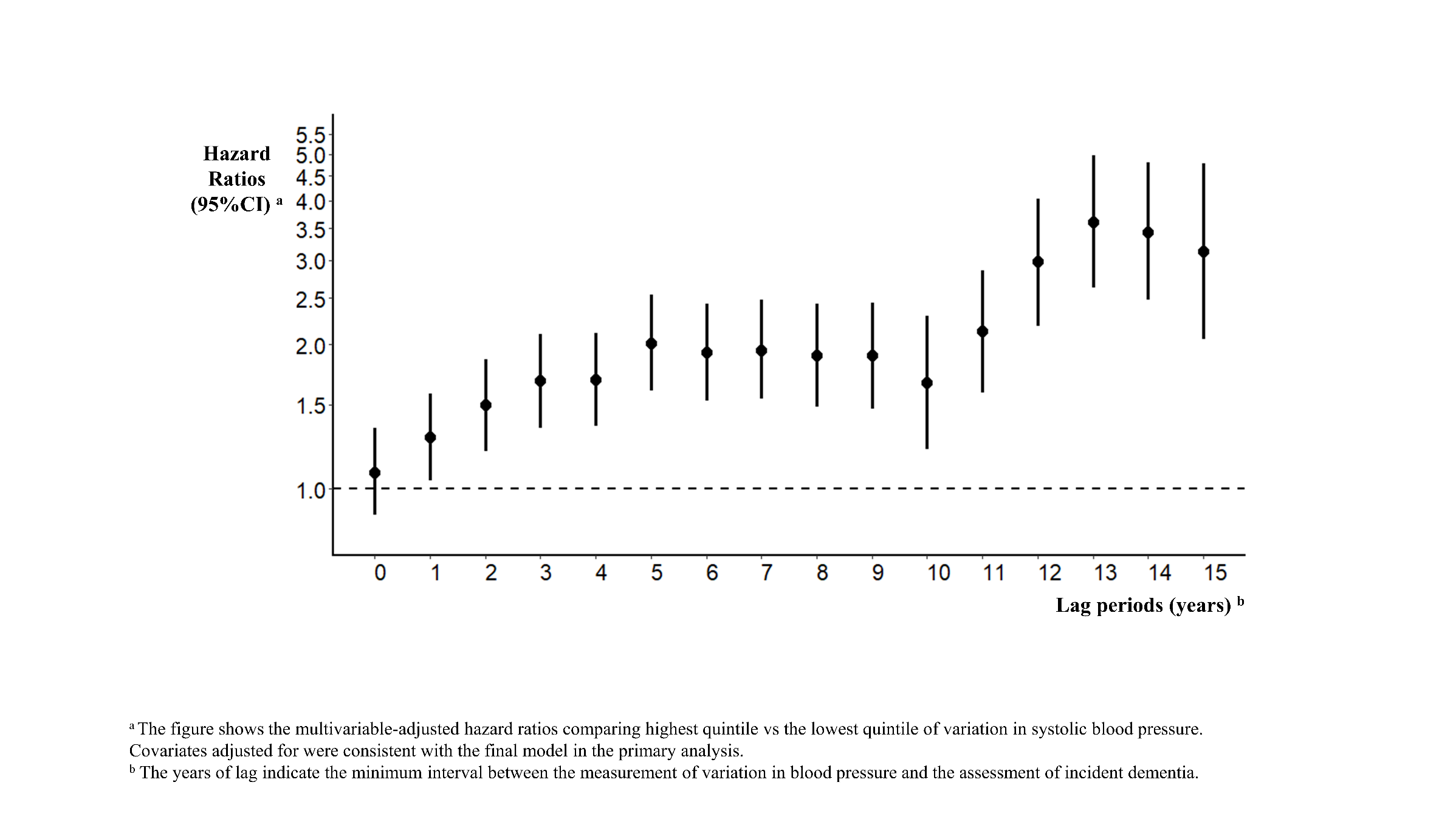


^a^ Multivariable-adjusted hazard ratio comparing the highest quintile versus the lowest quintile (<1.0% *v* ≥5.9% per year) of SBP variation. Covariates adjustment was consistent with the final model in the primary analysis.

^b^ The years of lag indicate the minimum interval between the measurement of the magnitude of SBP variation and the assessment of incident dementia.
