# Supplementary material for: Variation in blood pressure and long-term risk of dementia: A population-based cohort study
Source: PLoS Med. 2019 Nov 12;16(11):e1002933. doi: 10.1371/journal.pmed.1002933 (PMC6850672; doi:10.1371/journal.pmed.1002933)
Supplement: S3 Text — (DOCX) [file pmed.1002933.s004.docx]

**S3 Text. Detailed methodology**

**Covariate assessment**

We provide herein a detailed description on the assessment of the covariates adjusted in multivariate Cox models. Specifically, education was defined as low (primary education), intermediate (secondary general or vocational education), and high (higher vocational education or university) level. BMI (body mass index) was derived from weight and height measured at each visit using weight(kg) divided by height (m) squared and was categorized into four categories: underweight (<18.5), normal weight (18.5–25), overweight (25–30), and obesity (>=30 kg/m^2^). Smoking habits were categorized as current, former and never smoking. Total cholesterol and high-density lipoprotein (HDL)-cholesterol levels were acquired by an automated enzymatic procedure and was assessed as total cholesterol/HDL-cholesterol ratio (divided into quartiles). Hypertension was defined as a resting blood pressure exceeding 140/90 mmHg or the use of blood pressure lowering medication. Diabetes mellitus was defined as a fasting serum glucose level ≥7.0 mmol/L, non-fasting serum glucose level ≥11.1 mmol/L or use of anti-diabetic medication. History of cardiovascular disease was defined as any previous events of stroke, coronary heart disease (myocardial infarction and coronary interventions including percutaneous coronary interventions or coronary artery bypass grafts), heart failure, and atrial fibrillation, assessed via interviews and verified by reviewing medical records.^1^ Arterial stiffness was assessed by carotid-femoral pulse wave velocity at the 3^rd^ examination using an automatic device,^2,3^ which measured the time delay between the rapid upstroke of the feet of simultaneously recorded pulse waves in the carotid artery and the femoral artery. Pulse wave velocity index was calculated as the ratio between the distance and the foot-to-foot time delay and was expressed in meters per second.

**Inverse-probability weighted Cox models**

To minimize potential selection bias arising from the process of selecting individuals from overall study subjects into lag-specific analyses, we estimated hazard ratios from inverse probability weighted Cox models.^4^ Follow-up started from the 2^nd^ visit when the first measurement on blood pressure variation became available until the date of dementia diagnosis, date of death, date of loss to follow-up, or end of the study (01 January 2016), whichever came first. Therefore, individuals with a follow-up of <5 years were not included in the 5-year-lag analysis due to a lack of valid measurements on variation in blood pressure at time windows ≥five years prior to censoring. Likewise, individuals with a follow-up of <10 years were not included the 10-year-lag analysis, and so on. Inverse probability weights provide an alternative to reducing this issue.^5,6^ ^7^Specifically, the probability of being included in the period-specific analysis out of the overall study population (all 5,273 dementia-free participants) was estimated using logistic regression models. The nominator and denominator of the lag-specific weights were calculated separately. The weight nominator models modelled the probability of being included without considering covariates. The weight denominator models included the following covariates collected at the first examination: age, sex, education level, APOE genotype, baseline blood pressure level, smoking, alcohol drinking, BMI, lipid level, history of diabetes and history of cardiovascular disease. The final weights used in Cox proportional hazard models were stabilized weights truncated at the 1^st^ and 99^th^ percentiles and reflected the inverse of the probability of contributing to the lag-specific analysis. Robust sandwich estimator was used to allow for the dependence of weighted subjects.

**Reference**

1. Leening MJ, Kavousi M, Heeringa J, et al. Methods of data collection and definitions of cardiac outcomes in the Rotterdam Study. *Eur J Epidemiol* 2012; 27(3): 173-85.

2. Laurent S, Cockcroft J, Van Bortel L, et al. Expert consensus document on arterial stiffness: methodological issues and clinical applications. *European heart journal* 2006; 27(21): 2588-605.

3. Mattace-Raso FU, van der Cammen TJ, Hofman A, et al. Arterial stiffness and risk of coronary heart disease and stroke: the Rotterdam Study. *Circulation* 2006; 113(5): 657-63.

4. Mansournia MA, Altman DG. Inverse probability weighting. *BMJ* 2016; 352.

5. Cole SR, Hernan MA. Constructing inverse probability weights for marginal structural models. *American journal of epidemiology* 2008; 168(6): 656-64.

6. Hernan MA. How to estimate the effect of treatment duration on survival outcomes using observational data. *BMJ* 2018; 360: k182.

7. Hernan MA, Hernandez-Diaz S, Robins JM. A structural approach to selection bias. *Epidemiology (Cambridge, Mass)* 2004; 15(5): 615-25.
